# In anaerobic reactors the microbial community structure depends on feed type, with no “keystone” species tied to COD removal

**DOI:** 10.3389/fmicb.2025.1583463

**Published:** 2025-05-14

**Authors:** Olutooni B. Ajayi, William A. Arnold, Natasha Wright, Jeremy S. Guest, Paige J. Novak

**Affiliations:** ^1^Department of Civil, Environmental, and Geo-Engineering, University of Minnesota, Minneapolis, MN, United States; ^2^Department of Mechanical Engineering, University of Minnesota, Minneapolis, MN, United States; ^3^Department of Civil and Environmental Engineering, University of Illinois Urbana-Champaign, Urbana, IL, United States

**Keywords:** anaerobic digestion, feed type, high strength waste, two-stage systems, encapsulation, structure-function relationships, core communities

## Abstract

Two-stage anaerobic digestion (AD) systems provide treatment for high strength wastewater with high stability and performance. Encapsulation technology can intensify AD to facilitate the separation of the solids retention time from the hydraulic retention time (HRT), offering lower HRTs, smaller reactors, and high effluent quality. To support successful deployment, however, the encapsulated community must contain all the needed microorganisms for successful treatment and be flexible enough to treat a variety of wastewaters. Here, a two-stage system was investigated in which microbial cultures were enriched on various high-strength wastewaters in suspended flow-through systems to determine how feed type influenced performance and microbial community structure. The hypothesis was that specific genera, or so-called “keystone species” would positively correlate to organic carbon degradation for a given feed, enabling construction of a well-functioning community for encapsulation. Results showed that the number of total bacteria (as 16S rRNA gene copies) did not correlate to soluble chemical oxygen demand (sCOD) removal, indicating that the community structure and/or members were important for good performance. Results also showed that feed type strongly influenced carbon removal and microbial community structure for 1st-stage fermenting communities, but not 2nd-stage methanogenic communities. In this study, the “core” community members were defined as organisms common to all of either the 1st- or 2nd-stage reactors irrespective of the feed they received and were present in at least 50% of the samples throughout the entire experiment. “Unique” community members were specific to a single feed, and hence, only present in either the 1st- or 2nd-stage reactors receiving that feed. In both 1st- and 2nd-stage communities, only one core genera and no unique genera were positively and significantly correlated to sCOD removal. Verification experiments performed with encapsulated communities showed that organisms identified in flow-through system and correlated with carbon degradation, though not significantly, seemed to be important for performance. Our results suggest that one cannot construct a community containing specific populations in lieu of enrichment. Nevertheless, a single diverse encapsulated anaerobic community should provide good (>80%) carbon removal when fed a variety of influents, if time is provided for enrichment after deployment.

## 1 Introduction

Anaerobic digestion (AD) is a process used to treat wastewater with the high chemical oxygen demand (COD) loads associated with food and beverage industry wastewater (Bokhary et al., 2023). AD breaks down organic compounds via four distinct phases: hydrolysis, acidogenesis, acetogenesis, and methanogenesis, with CH_4_ and CO_2_ as end products (Uddin and Wright, 2023). AD can occur in both single- and two-stage configurations, though in a single-stage digester, process failure can be more common because the groups of organisms critical to degradation possess different growth rates and require different operational conditions for optimum performance (Meegoda et al., 2018; Onwosi et al., 2019). The separation of the acidogenic (1st stage) and methanogenic (2nd stage) reactors results in better stability with high organic loading and improved degradation and energy recovery (Fuentes et al., 2021; Rajendran et al., 2020). Research by Paranjpe and coworkers found a 41.9 and 9.8% increase in biogas yields and volatile solids (VS) removal efficiency, respectively, in a two-stage system compared to a single-stage digester co-digesting food waste and sewage sludge (Paranjpe et al., 2023). Another study observed that a two-stage system maintained its performance in terms of COD reduction and CH_4_ production across different hydraulic residence times (HRTs), whereas a single-stage comparison system declined in performance with varying HRT (Maspolim et al., 2015). Similarly, Schievano et al. (2014) reported the ability of two-stage systems to increase energy recovery from biomass digestion compared to single-stage AD systems. Because two-stage systems may require more space and capital equipment, it is important to ensure that they are operating as efficiently as possible if one is to reap the benefits of such a system.

In addition to adopting a two-stage system, different reactor configurations have also been used to optimize anaerobic treatment of high strength industrial wastewater, including continuously stirred-tank reactors (CSTRs), plug-flow reactors (PFRs), upflow anaerobic sludge blanket reactors (UASBs), and anaerobic membrane bioreactors (AnMBRs) (Nkuna et al., 2021; Rocha-Meneses et al., 2022). While most of these systems typically consume less energy for processing compared to their aerobic counterparts (typically 500–2,000 kWh/1,000 kg COD, or approximately 0.25–1.0 kWh m^−3^ for aeration) (Show and Lee, 2017), they can be characterized by high maintenance costs (Cowley and Brorsen, 2018). UASBs rely on microbial granulation, which is not suited to small-to-midsize applications where specialized personnel to maintain the system might not be available (Mariraj Mohan and Swathi, 2022). AnMBRs are characterized by high energy requirements, up to 5.68 kW h m^−3^, associated with maintenance operations such as the chemical cleaning and periodic backwashing to combat membrane fouling (Maaz et al., 2019; Shin and Bae, 2018). Some of these systems, such as CSTRs, also have the drawback of biomass washout. All of this creates a need to develop low-maintenance industrial anaerobic treatment systems, including two-stage systems, that are capable of low-energy separation of the biomass solids from the liquid, and thus, the solids retention time (SRT) from the HRT.

Encapsulation technology has garnered interest because of its ability to separate HRT from SRT, facilitate rapid system startup, and deploy specific desired organisms with a high degree of control (Chen J. et al., 2021; Chen S. et al., 2021; Lutz et al., 2023; Wang et al., 2021; Zhu et al., 2020). Encapsulation can also be used to successfully protect microbes from stressful environmental and operational conditions, allowing them to grow within an encapsulant (e.g., Wang et al., 2023) and improving effluent quality (Qin et al., 2020). Community shifts are also seen within an encapsulant, allowing for some degree of adaptation to occur over time (e.g., Xu et al., 2017; Yang et al., 2020). Past pilot-scale work performed with alginate encapsulants (Chen et al., 2022) demonstrated that for this technology to work, there is a need for (i) the microbial cultures to be enriched prior to encapsulation and (ii) an alternative encapsulant to alginate that is stable and durable. Fortunately, recent improvements in encapsulant chemistry have enabled the effective retention of organisms without encapsulant disintegration, for example in a polyethylene glycol encapsulant (Gutenberger et al., 2024). Nevertheless, because organisms are presumed not to invade encapsulants, the initial encapsulated community must contain all the organisms that will be needed for treatment over time. As a result, the identification of microbial populations capable of degrading particular types of waste (e.g., brewery, protein-rich or lipid-rich wastewater), a so-called “keystone” population or species, would enable the *a priori* selection of organisms for encapsulation and deployment, ensuring efficient and stable treatment over time and without the need for initial enrichment.

The literature is not clear as to how well the presence of a particular keystone organism correlates to the anaerobic degradation of certain wastes (Regueiro et al., 2015; Wang et al., 2020; Zhang et al., 2014). Therefore, the objective of this work was to enrich suspended microbial cultures in a flow through reactor on a variety of high-strength food and beverage wastewaters and determine the impact of the feed on soluble COD (sCOD) degradation and microbial community structure in 1st-stage fermenting and 2nd-stage methanogenic communities. An additional objective was to attempt to identify populations associated with good (80%) sCOD removal from certain feeds. Overall, we hypothesized that the feedstock type would strongly influence sCOD removal percent and the microbial community structure for 1st-stage fermenting communities, with identifiable keystone populations that correlate to excellent treatment potential for specific feed types. Conversely, we hypothesized that the 2nd-stage methanogenic communities would be similar regardless of the initial feed, with their relatively similar feeds (1st-stage effluents, including mostly fatty acids and alcohols) influencing the community in the 2nd-stage reactors as opposed to the initial 1st-stage feed. Finally, we tested our findings by operating reactors containing an encapsulated community to determine how the community shifted when enriched on three different feeds and whether the genera that increased in number could be tied back to those correlated to sCOD removal in the suspended flow-through reactors fed those same three feeds.

## 2 Materials and methods

### 2.1 Wastewater feed and inoculum

Five wastewaters were used as feedstocks in this study; these included three synthetic wastewaters (starch-rich, protein-rich, lipid-rich wastewater), one real brewery wastewater, and one real dairy wastewater. The synthetic starch-, protein- and lipid-rich wastewater were prepared using gelatin (Bovine Type B, Sigma-Aldrich), starch (Difco, BD), polysorbate 80 (Tween 80, Sigma-Aldrich), casamino acids (Fisher BioReagents) and yeast extract (Acumedia), added to the final concentrations shown in Supplementary Table S1. All recipes were modified from Zhu et al. (2020). The average COD of the three synthetic wastewaters was 8.8 g/L.

Brewery wastewater was collected approximately every 3 months in a 20-L plastic carboy from a holding tank at Fulton Brewery (Minneapolis, MN) and stored at 4°C. Dairy wastewater was collected approximately every 6 months in a 10-L plastic container from the concentrated stream of the Kemps Dairy’s (Farmington, MN) reverse osmosis unit and stored at 4°C until use. The average measured pH value of the starch-, protein-, and lipid-rich wastewater was approximately 6.5 and that of the brewery and dairy wastewater were 6.2 and 6.0, respectively. The initial sCOD of the brewery and dairy wastewater were approximately 20 g/L and 200 g/L, respectively. All experiments were performed with the brewery and dairy wastewater diluted to a final sCOD concentration of approximately 8 g/L. Anaerobic biomass used as inoculum was obtained approximately every 3 months from the digester at the Empire Water Resource Recovery Facility (Farmington MN) and stored at 4°C until use. For start-up, wastewater and inoculum were mixed in the flow-through reactors at a ratio of 2:1 by volume.

### 2.2 Biomass encapsulation

Cells were collected from biomass already enriched on high-strength wastewater feeds. Microbial cultures were encapsulated with polyethylene glycol (PEG) (30 mL PEG prepolymer solution containing 0.5 g of biomass per 50 beads) into bead-like structures, using the methods described by Gutenberger et al. (2024). Fermentative cells were encapsulated on the benchtop while the methanogenic cultures were encapsulated in an anaerobic glove bag (Coy). After encapsulation, the beads were rinsed with DI water, either on the benchtop (fermentative) or in a glove bag (methanogenic), prior to use.

### 2.3 Experimental design

Two different types of experiments were performed: (1) suspended growth flow-through reactors in which all five wastewaters were fed and (2) verification experiments in which encapsulated cultures were used and only three of the wastewaters were fed. The initial flow-through suspended growth reactors were used to test the hypotheses that feedstock type would strongly influence sCOD removal percent and the microbial community structure for 1st-stage fermenting communities but not for the 2nd-stage communities, with identifiable “keystone” populations correlated to sCOD removal from specific feed types. The reactors containing an encapsulated community were used to test the hypothesis that the community would shift when enriched on three different feeds and specific genera would increase in number based on trends observed in the suspended flow-through reactors fed those same three feeds.

#### 2.3.1 Flow-through suspended growth experiments

**Figure 1:**
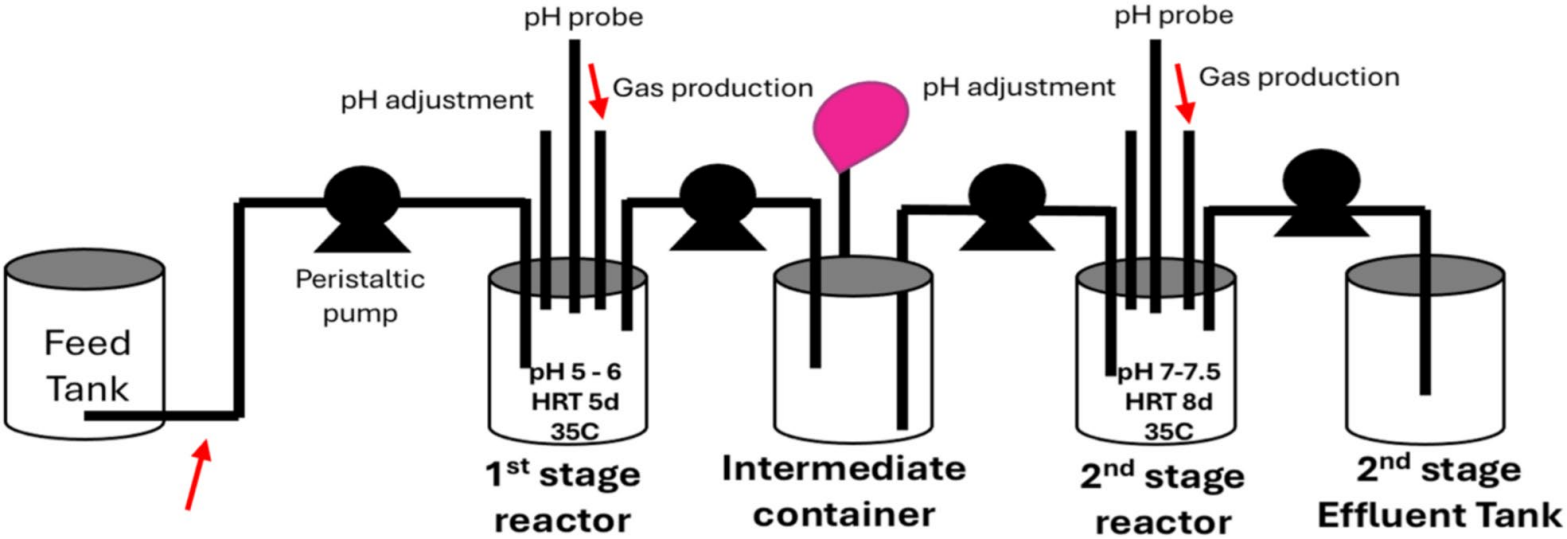
Schematic showing 1st-stage and 2nd-stage reactor set-up in which the reactors were connected in series. Red arrows indicate where samples were taken for analysis of sCOD, TSS, and VFAs. Samples were taken from the 1st-stage and 2nd-stage reactors for DNA extraction and biomass analysis.

Five sets of two 1-L glass reactors in series were built and set up with fermentative 1st-stage reactors followed by 2nd-stage methanogenic reactors (Figure 1). The reactors were constructed from screw-top glass jars with lids fitted with five ports to accommodate influent and effluent tubing, a pH probe, a pH adjustment line, and a gas line. Each of the five wastewater feeds was continuously stirred in feed tanks and pumped (Masterflex L/S Easy Load II pumps) into the 1st-stage reactors, which were operated at a pH of between 5 and 6 and a temperature of 35°C, adjusted with heating pads (Sunbeam) to improve enrichment. The pH was controlled using a Bluelab pH Controller & Auto Doser and 1 M HCl and 1 M NaOH. An intermediate container was placed between the 1st- and 2nd-stage reactors, where effluent from the 1st-stage was collected under nitrogen gas to manage the differences in HRT between the 1st- and 2nd-stage reactor. A nitrogen-filled balloon was attached to this container to maintain constant headspace pressure. The 2nd-stage methanogenic reactors were connected to the intermediate containers, each reactor receiving, as feed, the effluent from its paired 1st-stage reactor. These 2nd-stage reactors were designed to operate at a pH of 7–7.5 and at a temperature of approximately 35°C. Because of the complexity of these continuous-flow two-stage reactors, there were leaks in the system that prevented the quantification of biogas, including CH_4_. These five reactor sets were operated for 185 days.

The initial HRT of the 1st-stage reactors was 6 days but was decreased to 5 days on Day 106. The HRT was adjusted based on the success of fermentation observed in the reactor via volatile fatty acid (VFA) production. The initial HRT for the 2nd-stage reactors was 15 days, which was decreased to 8 days on Day 106 because sCOD removal was generally good, suggesting reasonable acclimation and growth. Although sCOD removal was generally good, TSS did decline periodically in the reactors, and reactors were reseeded with anaerobic biomass whenever TSS dropped below 1 g/L in the 1st-stage reactors and 2 g/L in 2nd-stage reactors or if sCOD in the reactor samples equaled the sCOD in the feed samples. The reactors were reseeded with one-third of the working volume as biomass. This reseeding took place approximately once a week for the 1st-stage reactors and every other week for the 2nd-stage reactors. After reseeding, the reactors were flushed with nitrogen gas.

Samples (7 mL) were collected in polypropylene centrifuge tubes twice a week directly from sampling ports on the feed line to the 1st-stage reactors and directly from the 1st- and 2nd-stage reactors to measure sCOD, TSS, and VFAs, and extract DNA for microbial community analysis. These were stored at −20°C prior to analysis. sCOD, VFA, and pH measurements for the entire system began on Day 55 of operation. This delay was required to allow the effluent COD from the 1st-stage reactors to become relatively constant before connecting the 2nd-stage reactors.

#### 2.3.2 Encapsulation experiment

A three-phase reactor system, identical to that described by Gutenberger et al. (2024), was used to set up a verification experiment to test how a single retained 1st-stage encapsulated community adapted when supplied with different feedstocks (Supplementary Figure S1). Feedstocks were selected based on similarities and differences in the suspended growth community structure and included the lipid-rich, protein-rich, and brewery wastewaters. Briefly, reactors were operated in three phases: an initial batch phase, a continuous plug-flow phase, and a final batch phase. The batch phases were set up in 120-mL serum bottles containing 60 mL of specific wastewater feeds. Encapsulated cultures were added to the reactors (15 beads/reactor) and VFA production and sCOD degradation were monitored for 5 days. Following the initial batch phase, the beads were moved from the batch reactors into 7-mL flow-through reactors. These flow-through reactors were supplied with the same feeds used in the initial batch phase. The flow-through reactors were operated for 2 weeks with an HRT of 45 min. After the 2-week flow-through period, the beads were again removed and transferred into a final batch phase reactor that was operated identically to the first batch phase reactor. Initial and final beads were collected from serum bottles and stored at −20°C until microbial analysis. All reactors were operated in triplicate.

### 2.4 Analytical methods

sCOD was quantified with the dichromate analysis method using COD reagent kits (HR + Hach COD kits, Hach, United States). For soluble COD, reactor samples samples were first filtered using 0.22 μm nylon filters (Restek), after which samples (250 μL) and 2,250 μL of deionized (DI) water were added to the reagents and digested for 2 h. sCOD standards (HACH, USA) were used to create calibration curves and handled identically to samples. Blanks were also analyzed, with 2.5 mL of DI water added to the reagents prior to digestion. The detection limit was 10 mg/L. VFAs were quantified with a high-performance liquid chromatograph (HPLC, Agilent 1100 Series) using an Aminex HPX-87H column (Bio-RAD) and a variable wavelength detector, as described previously (Chen et al., 2022). Samples were first filtered using 0.22 μm nylon filters and a volume of 120 μL was pipetted into LC vials and sealed with crimp lids. The instrument was operated at an injection volume of 10 μL, a flowrate of 0.6 mL/min, and a mobile-phase solution of 0.5 mM sulfuric acid. VFAs were detected at a wavelength of 210 nm. Standards from a 10 mM stock solution containing multiple VFAs were used to create external calibration curves for each set of analyses. DI water was used for blanks. The detection limit was 0.25 mM for all the acids.

### 2.5 Microbial community analyses

#### 2.5.1 DNA extraction

Samples (1.5 mL) of suspended cultures were collected from flow-through reactors, centrifuged (5,000 rpm for 10 min), and the supernatants discarded. The pellets were resuspended in 5% sodium dodecyl sulfate (SDS) lysis buffer, then subjected to three freeze (−20°C) and thaw cycles. Beads containing encapsulated cultures were collected and stored at −20°C. For DNA extraction they were first crushed inside a centrifuge tube using a glass rod (Supplementary Figure S2), then resuspended in lysis buffer and subjected to three freeze and thaw cycles. In both cases, DNA was extracted from the lysis buffer according to the manufacturer’s instructions using the MP BIO FastDNA Spin kit (Santa Ana, CA). DNA extracts were stored at −20°C until sequencing. The same DNA extracts were subsampled for Quantitative polymerase chain reaction (qPCR) analysis.

#### 2.5.2 qPCR

qPCR was performed on the extracted DNA samples to quantify the total biomass via 16S rRNA gene copy numbers (515F, GTGCCAGCMGCCGCGGTAA and 806R, GGACTACHVGGGTWTCTAAT) (Gohl et al., 2016). qPCR reactions were run in duplicate on a LightCycler^®^ 96 instrument (Roche Molecular Systems, Inc.). Each qPCR reaction assay (15 μL) contained 7.5 μL EvaGreen®, 0.45 μL each of the forward and reverse primers (10 μM), respectively, 0.25 μL bovine serum albumin, 5.35 μL DNase/RNase-free water and 1 μL DNA extract or standard. gBlock gene fragments containing the targeted genes (IDT, United States) were used as qPCR standards. The standards were serially diluted to create a calibration curve that ranged from 10^4^ to 10^9^ gene copies per reaction. The amplification efficiency for all reactions ranged from 94 to 99%. DNase/RNase-free water was used for the no template controls, which were included in each run. The concentrations of the genes were determined from the standard curve. The arithmetic mean of the duplicates was used to compute the cycle number (Cq) value for each sample. Detection limits were 1.6 × 10^5^ gene copies/mL reactor sample, which was lower than all measured samples.

#### 2.5.3 Sequencing

The 16S rRNA genes of the DNA extracts were amplified and sequenced at the University of Minnesota Genomics Center (UMGC) using paired-end sequences (2 × 300) on the Illumina Miseq platform. The sequencing data was filtered and rarefied via the Minnesota Supercomputing Institute (MSI) using the QIIME (qiime2/2019.10) pipeline. The sequences were clustered against one another at 99% similarity using the *de novo* method and classified into operational taxonomy units (OTUs) using the SILVA 138 rRNA database. The data presented in the study are deposited in the NCBI repository, accession number PRJNA1232696.

### 2.6 Data analysis

#### 2.6.1 Estimated absolute abundance analysis

The estimated absolute abundance of organisms was calculated as follows:


(1) $$N=[16S\;\text{rRNA copy number}\;(\text{molecules}/\mathrm{\mu L}\;\text{template})\times V_{t}]/V_{S}$$

(2) Estimated Absolute Abundance=N×RA

Where *N* is the normalized copy number from qPCR analysis (molecules/mL sample), *V_S_* is the volume of sample from the reactor (mL), *V_t_* is the volume of DNA template eluted from the sample (μL), and RA is the relative abundance of a particular population out of the entire number of sequences obtained from the UMGC sequencing data.

#### 2.6.2 Dissimilarity and statistical analyses

Non-multidimensional scaling (nMDS) was performed using the Bray-Curtis distance metric to determine the (dis)similarity of microbial communities in reactors enriched on different wastewater feeds. This analysis used rarified data and was executed in R version 4.2.1 using the vegan package version 2.6.4 (Oksanen, 2022). A four-dimensional solution (*k* = 4) was used to visualize the complex data structure so that samples that were closer together were more similar in composition, and vice versa. Several iterations (trymax = 100) were attempted and a stress level below 0.2 was considered acceptable for a reliable nMDS result. All statistical analyses on microbial communities were performed in R using the vegan package. In this study, the “core” community members were defined as the organisms that were common to all of either the 1st- or 2nd-stage reactors irrespective of the feed they received and were present (though in varying abundance) in at least 50% of the samples throughout the entire operational period. These communities were identified in R using non-rarified data. The “unique” community members were the organisms that were specific to a single feed, and hence, only present in either the 1st- or 2nd-stage reactors receiving that wastewater feed and were again computed in R using non-rarified data. A moving average was calculated by averaging the values of the current day and five preceding days to smooth variability in sCOD removal. Spearman correlations were performed between the system sCOD removal percentages and the estimated absolute abundance of the different members of the microbial communities to determine which organisms’ abundances were statistically correlated with sCOD removal percentages over 80% and therefore presumed to be indicative of good performance. Spearman correlations were used instead of Pearson’s correlations because the data exhibited both non-linearity and contained outliers. Correlations with a significance level > 0.05 were filtered out. Kruskal Wallis non-parametric tests were performed to compare average sCOD removal percentages across the 5 feed types and determine if they were statistically different during certain operational days. Mantel tests were performed to determine if there were correlations between the microbial community and operational factors. Shannon diversity index was calculated to determine the alpha diversity (richness and evenness) of the encapsulated microbial communities in the different reactors.

## 3 Results and discussion

### 3.1 sCOD removal

The start-up period for the reactors to stabilize, with respect to achieving consistent sCOD removal across the two stages and VFA production in the 1st-stage, was approximately 60 days; the 2nd-stage reactors were connected to the 1st-stage reactors in series on Day 55. VFAs were produced in the 1st-stage and, although gas could not be collected because of system leaks, degradation of sCOD to CO_2_ and CH_4_ was presumed, given the anaerobic nature and the loss of sCOD across the two stages.

**Figure 2:**
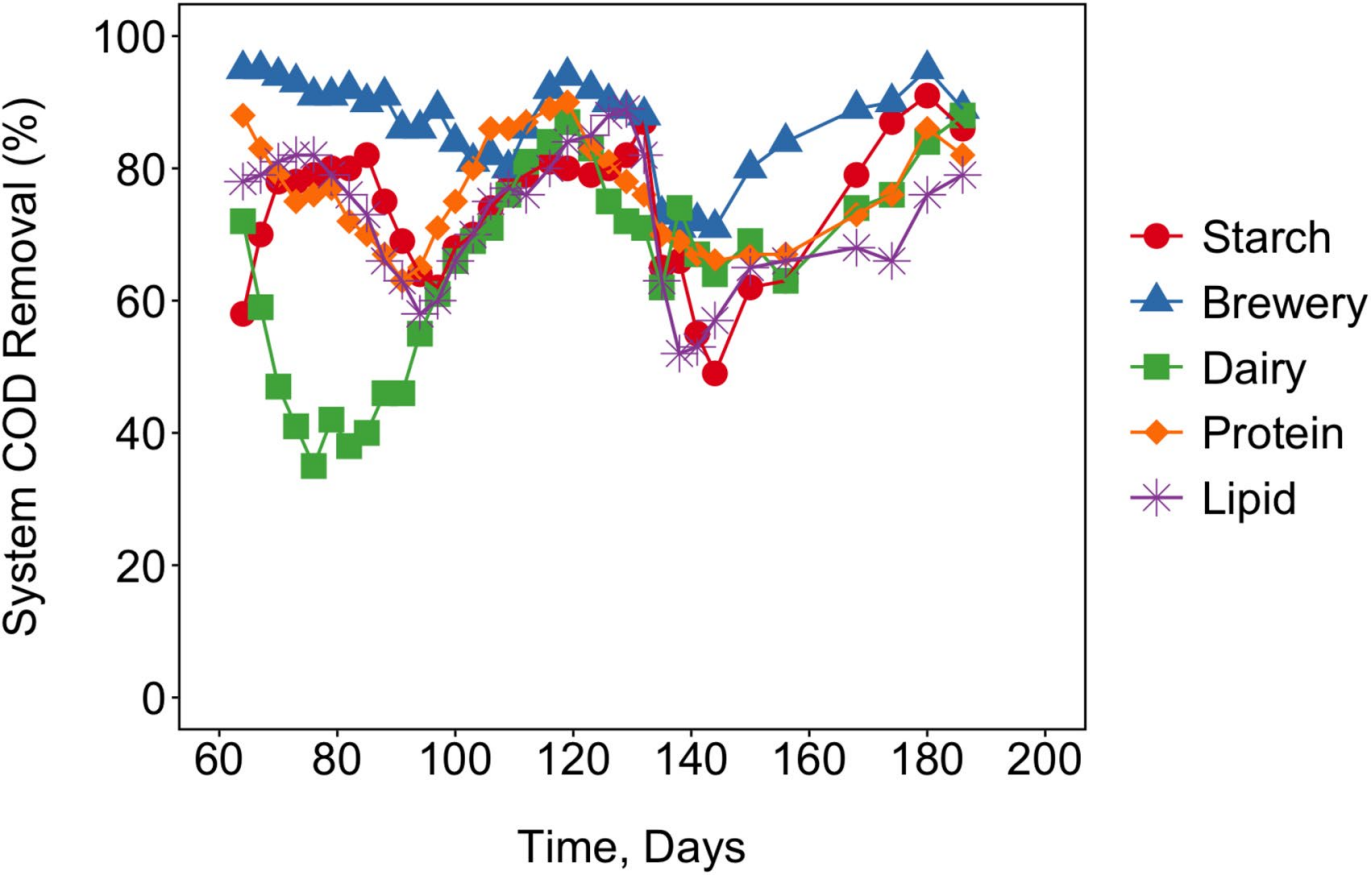
Percent soluble COD Removal (moving average) is shown over time across the combined 1st- and 2nd-stage reactors. Influent soluble COD was approximately 8.8 g/L for the synthetic lipid-rich, protein-rich, and starch-rich wastewater-fed reactors and approximately 8 g/L for the brewery and dairy wastewater-fed reactors. Connecting lines are to guide the eye.

sCOD removal between Day 60 and Day 192 was statistically influenced by feed type (*p* < 0.001), with a maximum total percentage of sCOD removal across the five different feed types of 79% (lipid-rich wastewater-fed reactors) to 96% (brewery wastewater-fed reactors) (Figure 2). Overall, the reactor receiving lipid-rich wastewater generally degraded the least sCOD compared to the reactors receiving the other feeds and the reactor receiving brewery wastewater degraded the most sCOD. Between Day 60 and Day 192 VFA production (Supplementary Figure S3) was also statistically influenced by feed type (*p* < 0.001) in all of the 1st-stage reactors. Despite these statistical differences, there were some similarities in the VFAs produced. The major VFA produced throughout all the 1st-stage reactors over the course of the experiment was propionate, with this VFA making up an average of 67% of the total VFAs measured across reactors and across time (Supplementary Figure S3). Isobutyrate was the second most observed VFA, averaging approximately 17% of the total VFAs across reactors and across time (Supplementary Figure S3). Acetate and isovalerate were periodically detected, with rare detections of butyrate and valerate (Supplementary Figure S3). There was a notable absence of VFA production in all reactors around Day 130 of operation, potentially attributed to biomass washout because of the reduction in HRT from 6 to 5 days. The production and distribution of different VFA profiles was likely a result of the differences in organic matter composition in the various feeds, as well as the mixed microbial cultures that became established in those reactors. The starch-rich wastewater-fed reactor exhibited much less VFA production. The reasons for this were unclear, although it could be that the starch feed was degraded more rapidly, resulting in degradation of the VFAs in the 1st-stage. The quantification of CH_4_ was not attempted because of leaks in the system. Nevertheless, the high sCOD removal efficiencies across the two stages indicated that CO_2_ and CH_4_ were the likely end products in the two-stage systems.

It has been observed that during the AD of food waste, the combined concentrations of propionate plus butyrate constituted approximately 15 and 70% of the total VFAs produced when the pH was maintained at 5 and 6, respectively (Wang et al., 2014). We saw similar trends with propionate, but in our systems isobutyrate was much more prevalent than butyrate, which was rarely detected. With respect to sCOD removal, McAteer and coworkers investigated the anaerobic treatment of dairy wastewater at 37°C in different reactor configurations, observing sCOD removal efficiencies of 83.2–92.9%, depending on the reactor used (McAteer et al., 2020). A comprehensive review by Holohan et al. (2022) reported a COD removal range of 59–91% during the treatment of lipid-rich wastewater using a lab-scale upflow anaerobic sludge blanket (UASB) reactor. Smetana and Grosser (2024) conducted a comprehensive assessment of a two-stage UASB treatment process applied to cassava wastewater, which served as a representative model for starch-rich wastewater; the study revealed substantial COD reductions of 86.4 and 93% at operational temperatures of 37°C and 55°C, respectively. Similarly, 90% COD removal was achieved with protein-rich wastewater using an anaerobic sequencing batch reactor (Deng et al., 2024).

Our results, as well as those from previous studies indicate the potential for successful anaerobic treatment of high strength industrial wastewater if biomass can be retained. Nevertheless, biomass washout occurred with each of our feeds, requiring regular biomass augmentation. This reseeding was followed by a temporary decline in sCOD removal, presumably while the community grew and again adapted to the given feed. The reactors in this study were specifically designed as flow-through systems with no biomass retention. This allowed us to monitor the communities’ adaptation patterns to the different feeds. Nevertheless, because of the periodic washout of biomass, it was thought that perhaps biomass concentration, rather than community structure, would be the most important variable controlling sCOD removal efficiency. There was, however, no discernible relationship between sCOD removal and the quantity of total biomass (as measured by 16S rRNA gene copies) in either the 1st- or 2nd-stage reactors (Supplementary Figure S4), as determined by the Spearman’s correlation test between sCOD removal and the quantity of total biomass (*p*-values for 1st and 2nd stage reactors were 0.75 and 0.53, respectively). This implies that the degradation of sCOD was influenced by specific members of the microbial community and/or the community structure, rather than only by the total quantity of biomass present.

### 3.2 Microbial community composition and adaptation

The varying sCOD removal percentages, statistically linked to feed type, also allowed for further probing into whether at any given time, community members, either in terms of number or composition, were correlated to sCOD degradation. Across the samples analyzed from the two stages, a total of 709 genera were identified after rarefying sequences to a depth of 3,000. The top 30 abundant genera were plotted for each feed type (Supplementary Figures S5, S6). Organisms observed included methanogens, acidogenic organisms, such as *Prevotella* (Inaba et al., 2020), and organisms commonly observed in other high strength waste treatment systems, such as *Candidatus* Cloacimonetes and *Bacteroidetes* (e.g., Suárez et al., 2018). Interestingly, methanogens did not dominate the community in the 2nd-stage reactors, though they were present in the top 30 genera. This was unexpected but could be a result of the flow-through nature of the suspended growth system and the propensity for slow-growing methanogens to wash out.

**Figure 3:**
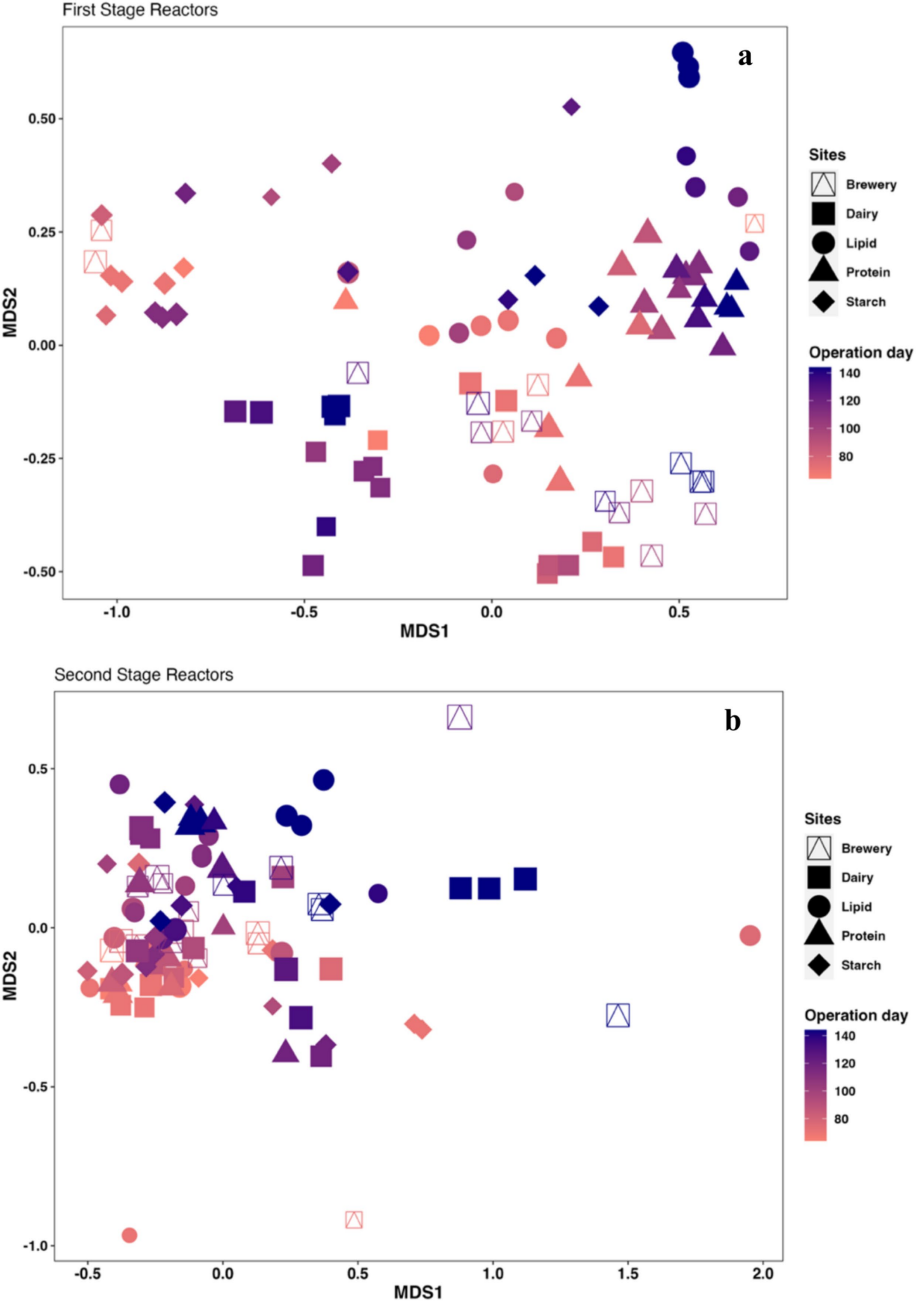
Non-multidimensional Scaling (nMDS) using Bray–Curtis dissimilarity, showing the differences in the microbial community structure in the different **(a)** 1st-stage and **(b)** 2nd-stage reactors with time. It can be seen that the 1st-stage communities separated from each other based on feed type. The 2nd-stage methanogenic communities also changed over time, but, with the exception of the dairy and lipid-rich wastewater-fed communities, did not visually separate by feed type.

Clear community shifts were observed over the course of the flow-through suspended growth experiment when reactors were supplied with different feeds (Supplementary Figures S5, S6), which was also visible from the nMDS plot generated using the Bray-Curtis distance matrix (Figure 3; Supplementary Figure S7). As the experiment progressed (colors in the figure changing from light pink to darker purple), samples taken from each of the 1st-stage reactors started to cluster into wastewater-specific groups, indicating the formation of distinct microbial communities in each reactor (Figure 3A). These community shifts noticeably occurred despite washout events and regular seeding. Some of the 1st-stage communities did cluster close to one another during the later periods of operation, which suggested that the communities (e.g., lipid-rich and protein-rich wastewater-fed communities) were more similar to one another in these reactors (Figure 3A). Despite the fact that the 1st-stage brewery wastewater-fed reactor had the most stable performance over time, the community structure changed over the entire sampling period (Figure 3A). This type of pattern has been observed by others as well (Fernández et al., 1999) and could indicate that a variety of hydrolytic, acidogenic, and acetogenic communities, characterized by a diversity of members, are capable of degrading brewery wastewater rather than a highly specialized community. Complicating this analysis, however, is the fact that the brewery wastewater itself is likely to be biologically active (Chen J. et al., 2021; Chen S. et al., 2021), which may have prevented the development of a steady community structure.

In the 2nd-stage methanogenic reactors (Figure 3B; Supplementary Figure S7), the community structure of the samples taken on Day 60 from all the reactors clustered tightly (light pink symbols), after which some spread in the community structure was observed over time (Figure 3B; Supplementary Figure S7), which is likely to be reflective of the slightly different VFA profiles in the 1st-stage effluents over time (Supplementary Figure S3), but generally shifted less, given the relatively similar feeds of fermented 1st-stage effluent.

Statistical correlations between operational parameters and the microbial community structure were also investigated. The Mantel test revealed that feed type significantly influenced the 1st-stage microbial community structure (*r* = 0.3088, *p* = 0.001), despite regular reseeding, but not the 2nd-stage community structure (*r* = 0.011, *p* = 0.362). This supports the idea that the communities adapted relatively rapidly to the feed type, and it also supports the validity of trying to identify organisms associated with high sCOD removal in each of the reactors. The results also suggest that the 1st-stage effluents did not play a strong role in shaping the community structure in the 2nd-stage reactors. We had hypothesized that the 2nd-stage reactor communities would be more similar to one another, given their feed of fermented/degraded 1st-stage effluent, and this hypothesis was generally supported by the sequencing and statistical data.

### 3.3 Correlations between sCOD removal and estimated absolute abundance of core and unique communities

As mentioned above, the “core” community members were defined as the organisms that were common to all of either the 1st- or 2nd-stage reactors irrespective of the feed they received and were present (though in varying abundance) in at least 50% of the samples throughout the entire operational period. These communities were generated from non-rarified data, and absolute abundance for each taxon was estimated using Equations 1 and 2. This resulted in the identification of 972 genera. The “unique” community members were the organisms that were specific to a single feed, and hence, only present in either the 1st- or 2nd-stage reactors receiving that wastewater feed.

Across all feed types, a total of 9 core phyla (including an unidentified phylum) were present in the fermentative 1st-stage reactor samples and 13 core phyla were present in the 2nd-stage methanogenic reactor samples (Supplementary Figure S8). For the 1st-stage reactors, the most abundant core phyla were *Desulfobacterota* (22%), *Deferribacterota* (19%), *Bacteroidota* (18%), and *Proteobacteria* (17%). For the 2nd-stage reactors, the most abundant core phyla were: *Campilobacterota* (24%), *Cloacimonadota* (16%), *Synergistota* (14%), *Proteobacteria* (5%), and *Desulfobacterota* (3%). Similar core communities observed in both stages could be a result of the same mixed culture being used to seed the reactors initially and upon washout. Nevertheless, in the 2nd-stage communities, additional distinct phyla were also identified, as shown below. It is notable that methanogens were present only in the 2nd-stage core phyla *Halobacterota* (5%).

On the genus level, 24 and 42 core genera were identified in the 1st-stage and 2nd-stage reactors, respectively, including uncultured and unidentified organisms (Supplementary Table S2). Of those, only 1 genus (*Candidatus* Cloacimonas*, ρ* = 0.72) had a significant and positive correlation with sCOD removal (Dairy, 1st-stage); all other significant correlations were negative (Table 1), demonstrating inverse correlations (e.g., as the population numbers decreased, sCOD removal increased). This suggests that core organisms may be largely important for the stability and function of the microbial community in general, but not specifically required for the degradation of a particular feed type. This is supported by the fact that several of the organisms present in the 1st-stage reactors, for example, *Lactococcus* spp., *Candidatus* Cloacimonas spp., *Pseudomonas* spp., and *Seleniivibrio* spp., are known to degrade a variety of wastewater types, including dairy wastewater (McAteer et al., 2020), black water (Dang et al., 2022), and municipal wastewater (Odjadjare et al., 2012) and are more indicative of a complex community capable of degrading a variety of COD types. In the 2nd-stage, identified core organisms, *Arcobacter*, *Dechlorobacter*, and *Bacteroides*, are also known for the degradation of complex organic matter in anaerobic systems, facilitating interspecies electron transfer for CH_4_ production and generating VFAs during the early stages of methanogenesis (Kristensen et al., 2020; Zhou et al., 2023; Borth et al., 2022). These results are consistent with those that showed that the most abundant genera were those commonly observed in other high strength waste treatment systems (e.g., Suárez et al., 2018). Nevertheless, in the 1st-stage reactors the community structure was statistically correlated with feed type, suggesting that although single keystone organisms from the core genera could not be linked to sCOD degradation in a particular feed, enrichment of a community into a particular structure was important for performance.

**Table 1:** The 1st- and 2nd-stage core and unique genera that statistically (*p* < 0.05) correlated with soluble COD removal based on their estimated absolute abundance in reactors fed different wastewater based on Spearman correlations.

Waste type	Core genus (rho)		Unique genus (rho)	
	1st-stage	2nd-stage	1st-stage	2nd-stage
Starch-rich	*Lactococcus* (−0.88)	*Arcobacter* (−0.77) *Dechlorobacter* (−0.53)	*Meniscus* (−0.74)	
Brewery				*Sva0996_marine_group* (−0.62) *Gemmata* (−0.62)
Dairy	*Candidatus* Cloacimonas (0.72) Unidentified (−0.63)	*Bacteroides* (−0.70)	*Psychrobacter* (−0.58)	
Protein-rich	*Pseudomonas* (−0.56) *Seleniivibrio* (−0.63)		*Shuttleworthia* (−0.56) *Aquamicrobium* (−0.55)	
Lipid-rich	–	–	–	–

Note that all but one correlation (*Candidatus* Cloacimonas) were negative.

Unique genera were also observed in all the reactor systems. In the 1st- and 2nd-stage reactors, respectively, a total of 49 and 53 unique genera were found in the starch-rich wastewater-fed reactors, 11 and 39 in the brewery wastewater-fed reactors, 28 and 27 in the dairy wastewater-fed reactors, 60 and 17 in the protein-rich wastewater-fed reactors, and 28 and 26 in the lipid-rich wastewater-fed reactors. With respect to correlations between unique genera and sCOD removal, five unique genera had significant correlations with sCOD removal, although they were all negative (Table 1). Similar to the patterns observed with the core genera, the unique genera correlated with sCOD removal were connected in the literature to more general COD removal rather than the degradation of specific compounds (Świątczak et al., 2017; Alalam et al., 2021; Honerlagen et al., 2022; Zafar et al., 2023). The fact that no unique genera correlated positively to sCOD degradation in the 1st- or 2nd-stage flow-through suspended growth reactors suggests that there are no keystone organisms from the unique genera that are responsible for excellent sCOD removal; indeed, the retention of a more complex community that develops over time appears to be most critical for treatment efficacy.

With respect to community diversity, the Shannon index values for the 1st-stage reactors ranged from 6.3 to 8, while those for the 2nd-stage reactors ranged from 5.5 to 8.4. The Shannon index, however, did not correlate to sCOD removal. This likely suggests that the reactors, as noted, contained a sufficiently diverse community to enable robust food- and beverage-wastewater sCOD degradation, with no advantage observed for additional diversity. If the communities had been simpler, it is possible that the Shannon index would have been more indicative of sCOD removal potential.

### 3.4 Experimental verification of results with an encapsulated culture

The community from the 1st-stage reactor fed lipid-rich wastewater was encapsulated to achieve near complete biomass retention, sequenced after encapsulation, then exposed to different wastewater feeds (lipid-rich, protein-rich, and brewery wastewater) to determine how these initial fermentative encapsulated communities would perform and adapt over time when the biomass was retained but fed different wastewaters. This community was selected because it was similar in structure to the protein-rich wastewater-fed reactor community but different from the brewery wastewater-fed community, providing us with a community that should be capable of adapting to at least one of the other wastewater feeds relatively well, though perhaps not to all three. Our hypothesis was that 1st-stage community members important for total sCOD removal from a given feed would increase in abundance during the adaptation period.

**Figure 4:**
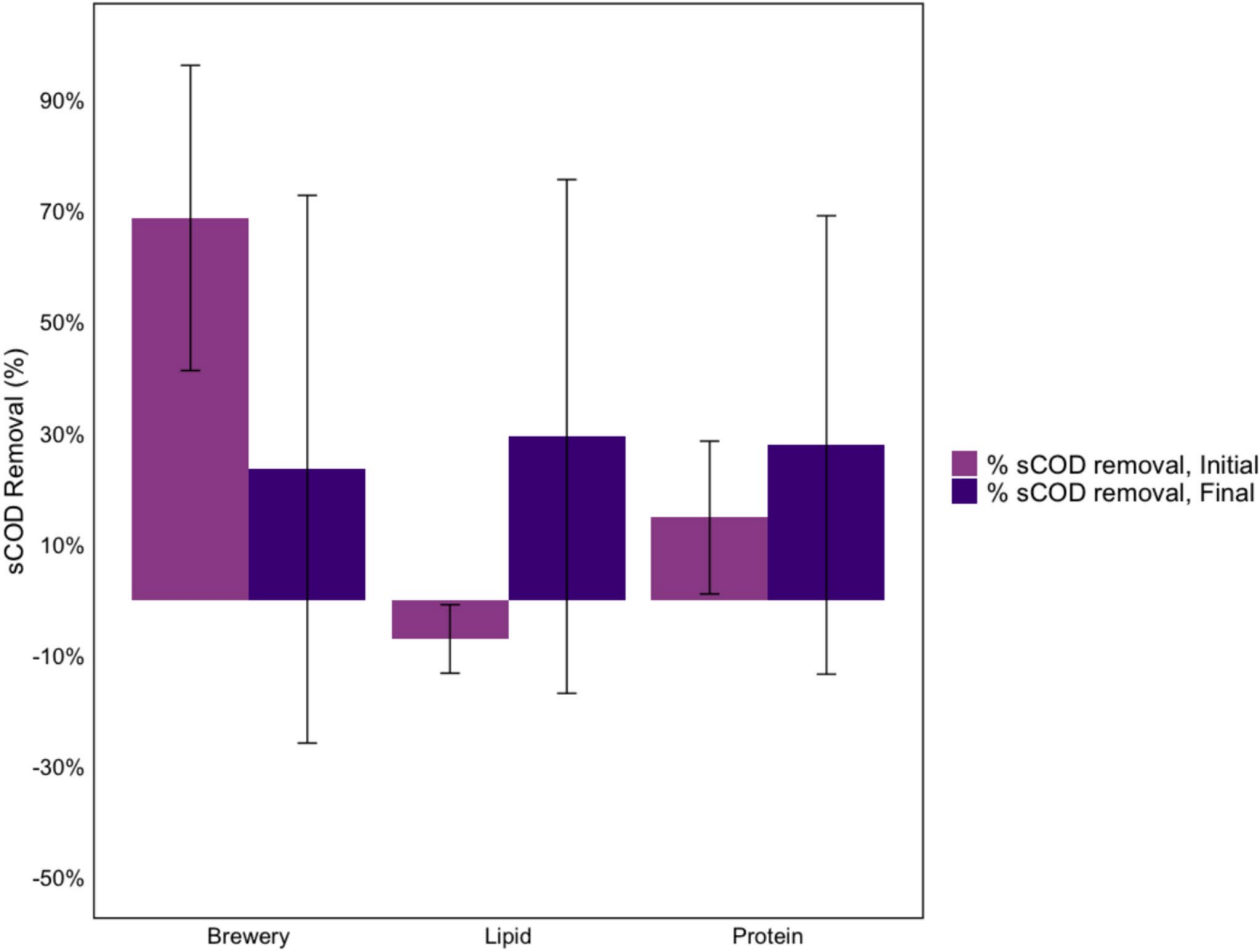
Soluble COD removal over 5 days in the initial and final batch phase experiments containing encapsulated lipid-adapted cultures fed lipid-rich, protein-rich, and brewery wastewater.

**Figure 5:**
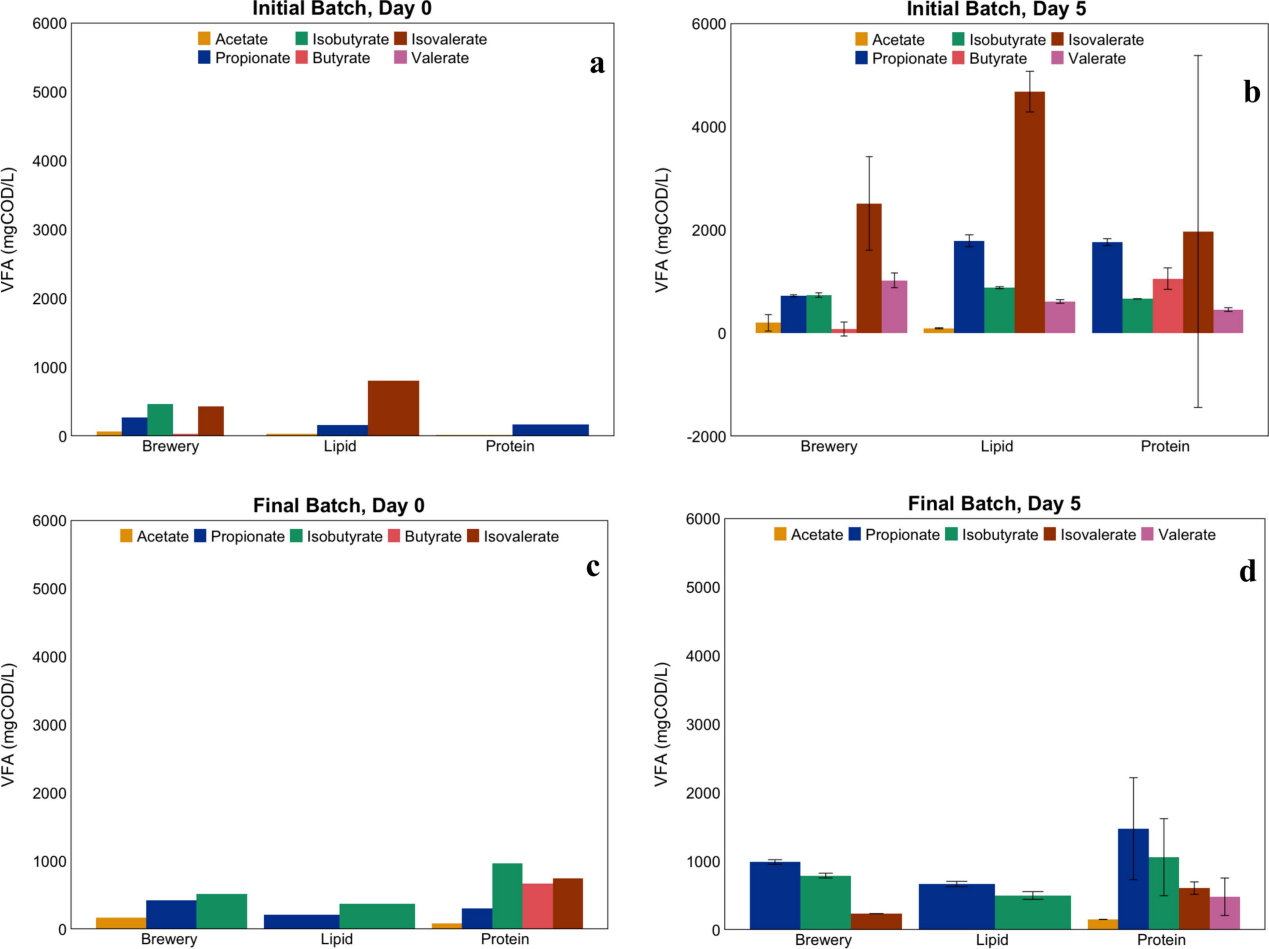
VFA concentrations on Day 0 (panels **a,c**) and Day 5 (panels **b,d**) in initial (panels **a,b**) and final (panels **c,d**) batch phase experiments with encapsulated lipid-adapted cultures fed lipid-rich, protein-rich, or brewery wastewater. Note that the axes for the plots from the initial and final batch phase experiments are on different scales. Error bars are not given for the Day 0 samples, as only one sample of the feed (common to all replicates) was taken for analysis.

Similar performance in terms of sCOD removal (Figure 4) and VFA production (Figure 5), was observed in the initial batch reactors fed protein-rich and lipid-rich wastewater, which is in line with the observations made from our initial flow-through suspended growth system, in which similar communities developed over time in those 1st-stage reactors (Figure 3). VFA production in the batch reactors fed brewery wastewater (containing the encapsulated lipid-adapted culture) occurred to a lesser extent during the initial batch phase. sCOD removal percentages, expected to be low, as the experiment was performed with 1st-stage fermentative communities, were different in all three systems, with the highest sCOD removal in the brewery wastewater-fed batch reactor and poor sCOD removal in the lipid and protein-rich wastewater-fed batch reactors (Figure 4). This may indicate that sCOD was degraded beyond VFAs in the brewery wastewater-fed reactors, with some methanogenesis occurring. After adaptation, sCOD removal improved for the systems fed lipid- and protein-rich wastewater but declined for those fed brewery wastewater. VFA production declined in all three systems, again suggesting degradation beyond fatty acids.

The communities in the encapsulants all changed during enrichment and adaptation, as expected. The encapsulated communities all originated from the 1st-stage suspended growth flow-through lipid-rich wastewater fed reactor; therefore, we analyzed for the core (24) and unique (28) genera originally present in that community. The initial communities in the encapsulant and those present after adaptation to the three different feeds (lipid-rich, protein-rich and brewery wastewater) were analyzed and compared to the core and unique genera observed in the suspended growth flow-through reactors to determine whether particular organisms were likely to be responsible for good sCOD removal from the different feeds.

**Figure 6:**
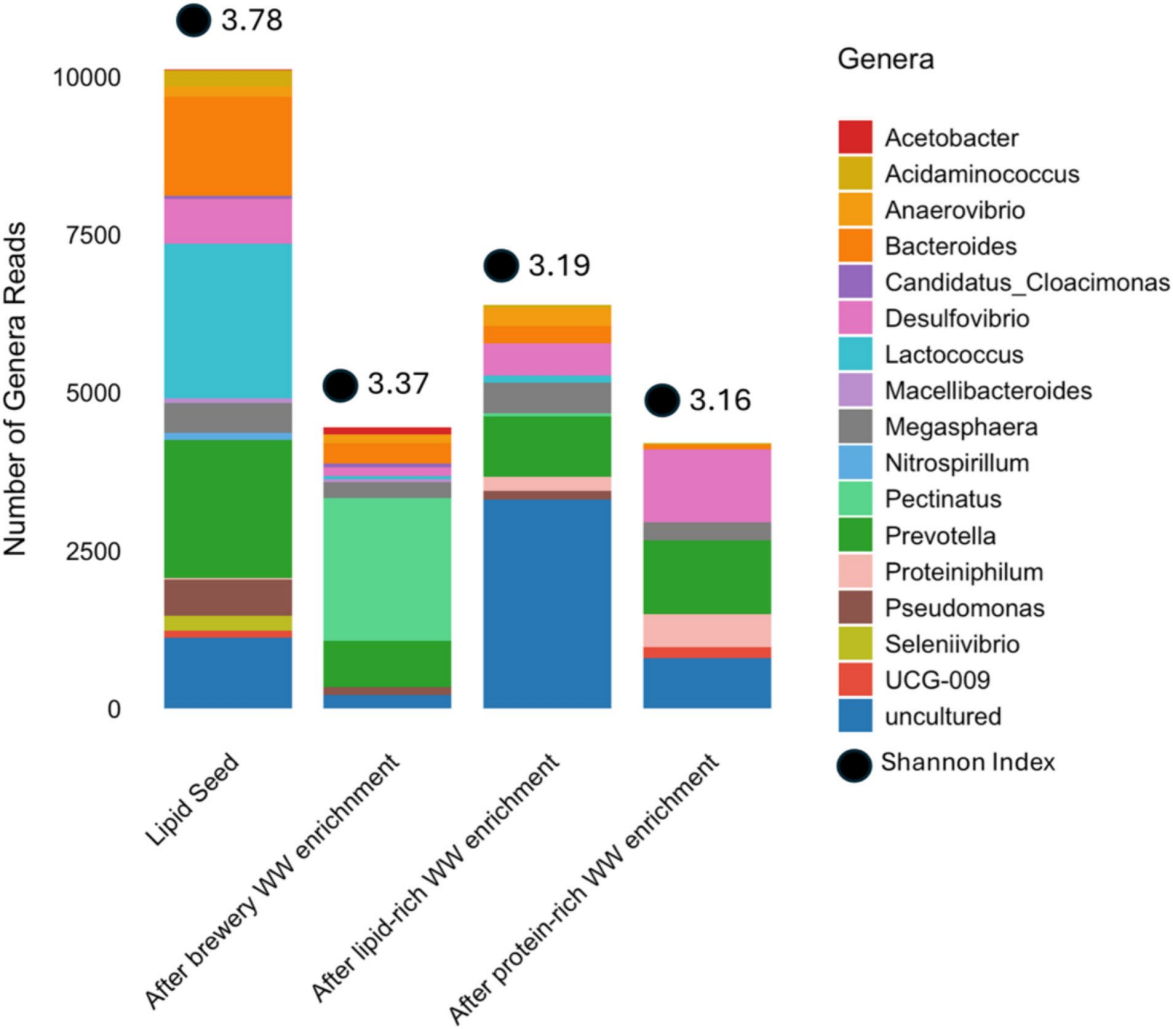
Abundance of all core genera in the encapsulated culture before and after enrichment.

**Figure 7:**
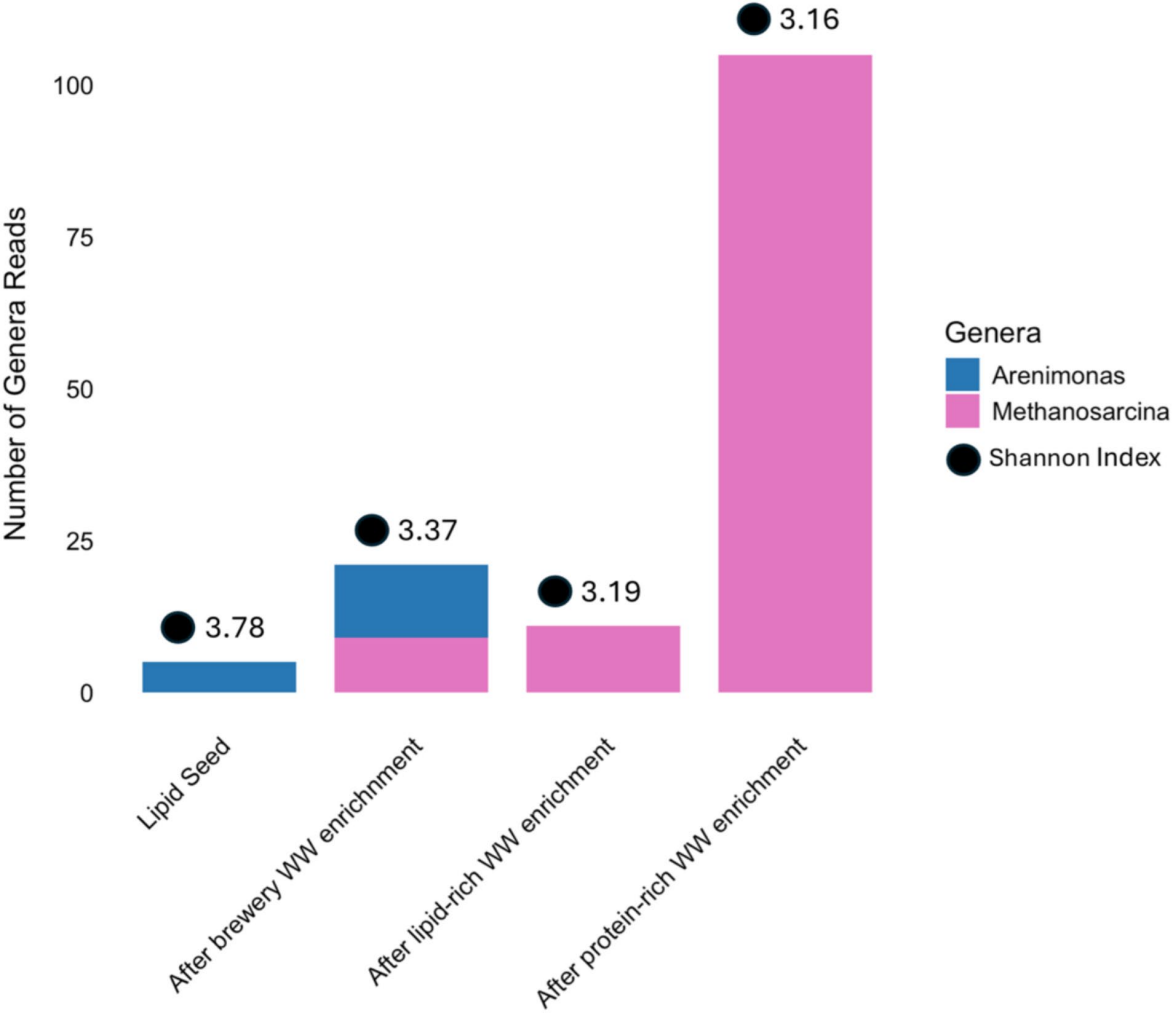
Abundance of genera unique to brewery feedstock in the encapsulated culture before and after enrichment.

After encapsulation but prior to adaptation, a total of 16 genera that were previously found in the core community of the 1st-stage suspended flow-through reactor systems were present in the encapsulated community (Supplementary Table S2). Interestingly, after this encapsulated community went through 2 weeks of adaptation on the three different feeds, most of these core genera decreased in abundance (Figure 6), including *Seleniivibrio*, which was entirely absent after enrichment/adaptation, and *Pseudomonas*, which declined in relative abundance in the encapsulants enriched on brewery and lipid-rich wastewater and was absent in those enriched on protein-rich wastewater (Figure 6). One genus present in the core community of the flow-through suspended growth reactors but initially absent in the encapsulated community, *Pectinatus*, was detected after enrichment on brewery and lipid-rich wastewater (Figure 6). Indeed, upon enrichment on brewery wastewater, the genera *Acetobacter* and *Pectinatus* increased in abundance (13–114 and 0–2,270), suggesting their potential importance in degrading brewery wastewater. *Acetobacter* and *Pectinatus* have been observed to contribute the spoilage of beer during brewing (Obi, 2017; Rodríguez-Saavedra et al., 2021), which suggests that they may be important for the degradation of organic compounds specific to brewery wastewater, including ethanol. Upon enrichment on protein-rich wastewater, *Desulfovibrio*, *Proteiniphilum*, and *UCG-009* increased in abundance, suggesting their importance for protein-rich wastewater treatment (Figure 6). These organisms have been observed to be important in amino acid degradation in different wastewaters (Deng et al., 2022; Feng et al., 2023; Hernandez-Eugenio et al., 2000). After enrichment on lipid-rich wastewater, *Anaerovibrio*, *Pectinatus*, *Proteiniphilum* and an uncultured genus all increased in abundance (Figure 6). The presence of *Proteiniphilum*, observed in both lipid- and protein-enriched communities, supports the microbial community analysis described earlier (Figure 3) where similar community structures appeared to develop in the reactors fed lipid- and protein-rich wastewater. *Prevotella* decreased in abundance by 56% (Figure 6). Performance (sCOD removal and VFA production) declined over the same time period, indicating that this organism might be important for lipid-rich wastewater treatment. Others have observed that *Prevotella* increased in abundance when lipid wastewater was treated with concomitant propionate production (Xu et al., 2023). In our experiment, propionate production declined by about 50% between the initial batch phase and the final batch phase (Figure 5), which is consistent with the results of Xu et al. (2023). With respect to the unique organisms originally identified in the flow-through suspended growth reactors, all except *Arenimonas* and *Methanosarcina* were missing in the encapsulant (Figure 7) before and after enrichment, again suggesting that the unique organisms identified in the flow-through suspended growth reactors were not well-maintained during encapsulation and may not be critical for the fermentation of these three wastes. Finally, over the enrichment period, the diversity of the community decreased. This was perhaps expected, as performance indicators also generally declined (Cira et al., 2018). This again supports the idea that a general and diverse microbial community is perhaps the most important indicator of treatment potential, along with the ability to enrich a given community on a specific waste.

## 4 Environmental and industrial significance

Several important conclusions can be drawn from this research. First, we observed that in a two-stage system, feed type strongly influenced sCOD removal and microbial structure for 1st-stage fermenting communities but not for 2nd-stage methanogenic communities. This is likely because the feedstock to the 2nd-stage reactors was fermented effluent and therefore, essentially served as a 6^th^ type of feed (“fermented effluent”) rather than 5 more unique feeds. Second, the overall biomass concentration did not correlate to sCOD removal across the two reactor stages, pointing to the importance of an enriched community, particularly an enriched fermentative community capable of breaking down particular feedstocks into VFAs, for effective sCOD removal. In addition, despite the fact that feed type had a strong influence on the overall community structure, only one organism in both the core and unique communities of both reactor stages correlated positively to sCOD removal (*Candidatus* Cloacimonas, 1st-stage, dairy wastewater-fed). This indicated that particular genera, or so-called “keystone organisms” are not required for effective sCOD removal from different wastewater feeds, but rather, a single initial complex culture can adapt to different wastewater feeds relatively quickly. Because the type of feed influenced community structure, prior enrichment on a given type of waste should improve treatment. This was supported by the experiments containing encapsulated communities adapted to lipid-rich wastewater, wherein differential enrichment was observed during exposure to lipid-rich, protein-rich, or brewery wastewater, clearly indicating the need for enrichment and the influence of feed type on fermentative community structure, but again with no indication of the presence of keystone organisms.

With respect to industrial significance, enrichment was important for performance, including enrichment within the encapsulant itself. Because no keystone genera seemed to exist, it appears that enrichment cannot be avoided, at least in 1st-stage fermentative reactors, via the construction of a simple community consisting of specific organisms. This is important for the deployment of an encapsulation system in which fast start-up is of great interest: the deployment of a complex, and ideally pre-enriched encapsulated community is likely to be much more successful in terms of rapid start-up and good treatment performance than deployment of a constructed and simpler community containing particular organisms. It also suggests that the use of encapsulated communities can be applied more widely, as an initial complex community will continue to adapt once deployed.

## Data Availability

The data presented in the study are deposited in the NCBI repository, accession number PRJNA1232696 (https://www.ncbi.nlm.nih.gov/bioproject/PRJNA1232696).
