# Supplementary material for: In anaerobic reactors the microbial community structure depends on feed type, with no “keystone” species tied to COD removal
Source: Front Microbiol. 2025 May 14;16:1583463. doi: 10.3389/fmicb.2025.1583463 (PMC12116630; doi:10.3389/fmicb.2025.1583463)
Supplement: Supplementary file 1 [file Data_Sheet_1.PDF]

Supplementary Material

In anaerobic reactors the microbial community structure depends on feed type, with no "keystone" species tied to COD removal

Olutooni B. Ajayi[1], William A. Arnold[1], Natasha Wright[2], Jeremy S. Guest[3], Paige J. Novak[1*]

[1]Department of Civil, Environmental, and Geo- Engineering, University of Minnesota, Minneapolis, MN, USA

[2]Department of Mechanical Engineering, University of Minnesota, Minneapolis, MN, USA

[3]Department of Civil & Environmental Engineering, University of Illinois, Urbana-Champaign, Urbana, IL, USA

*** Correspondence:**

Paige J. Novak

novak010@umn.edu

Supplemental Methods

Table S1. Synthetic wastewater recipes used in the experiment

	Quantities (g) added to water to a final volume of 1 L						
Wastewater Type	Polysorbate 80	Gelatin	Meat Extract	Casamino Acids	Starch	Yeast Extract	Rapeseed Oil
Starch-Rich	0.96	1.5	-	0.08	4.0	0.08	-
Protein-Rich	0.96	3.5	1.0	0.08	1.0	0.08	-
Lipid-Rich	0.96	2.32	-	0.08	1.18	0.08	2.0

a

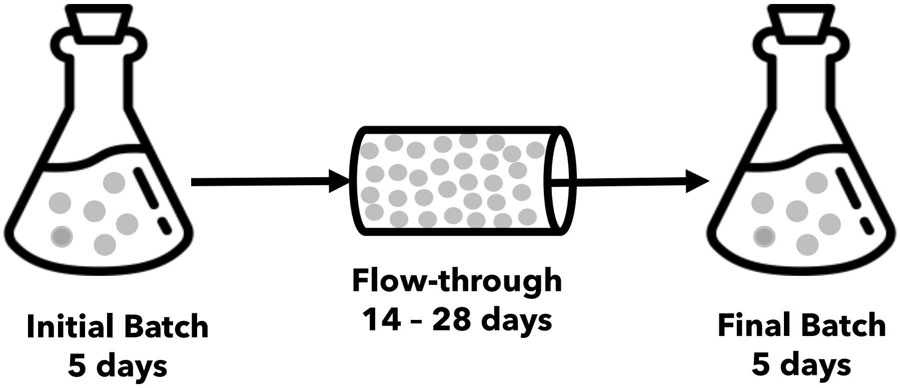

b

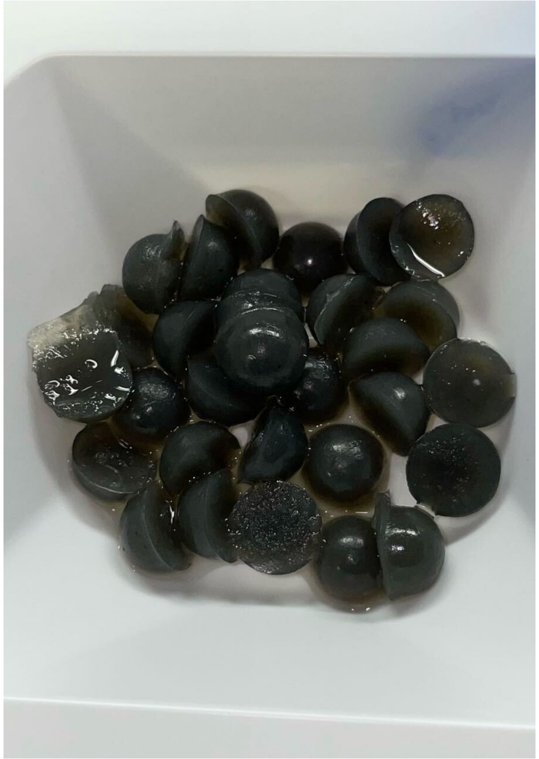

Figure S1. (a) Schematic showing the three-phase system including an initial batch reactor, a flow-through reactor and final batch reactor. After 5 days, beads were removed from the batch reactor and placed in the flow-through reactor. After 14 days in the flow-through reactor, they were returned to batch reactor to assess changes in VFA production and sCOD degradation over a final 5-day batch period. **(b)** Image of beads made from 2nd-stage methanogenic cultures.

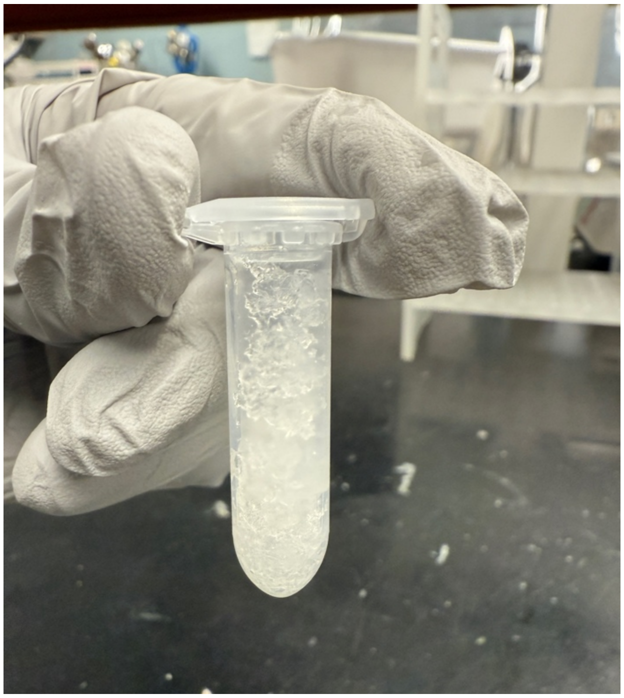

Figure S2. Photo of crushed bead used for DNA extraction.

Supplemental Results

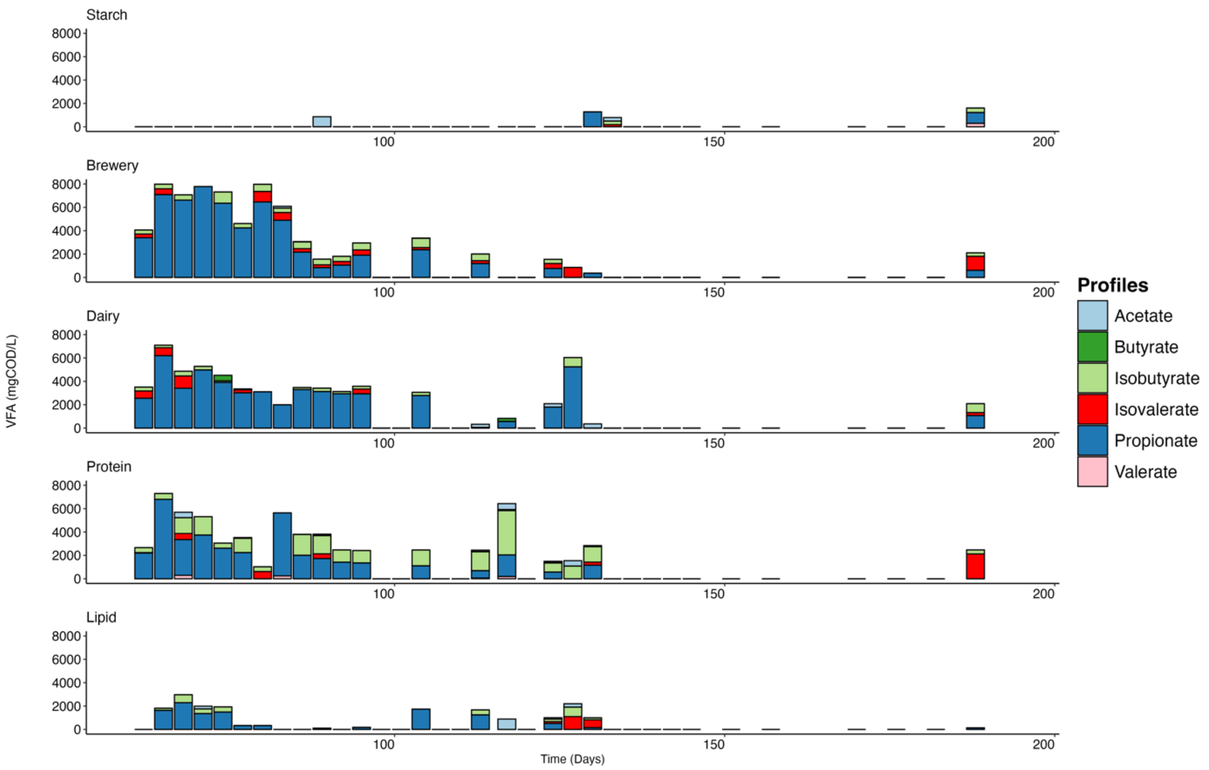

Figure S3. VFA production over time in the 1st-stage reactors fed different wastewaters. The wastewater feed type is shown for each panel. On the days with no results shown, no VFAs were detected in samples.

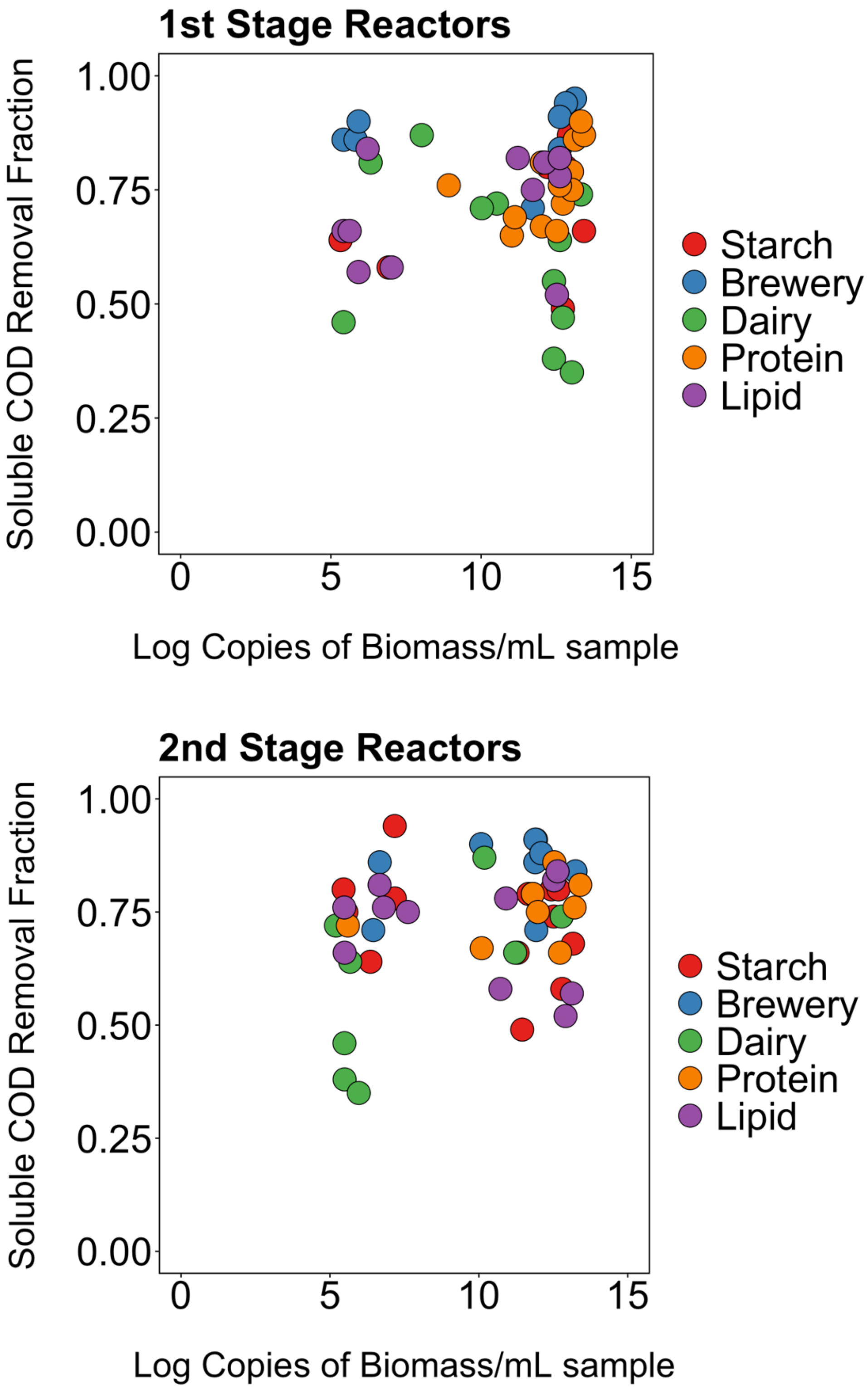

Figure S4. Total two-stage system COD removal as a function of total biomass concentrations (measured as total 16S rRNA gene copies/mL reactor) present in the 1st-stage and 2nd-stage reactors. Correlations between COD removal and log copy of biomass/mL reactor were not statistically significant, with p-values of 0.75 for the 1st-stage, 0.53 for the 2nd-stage.

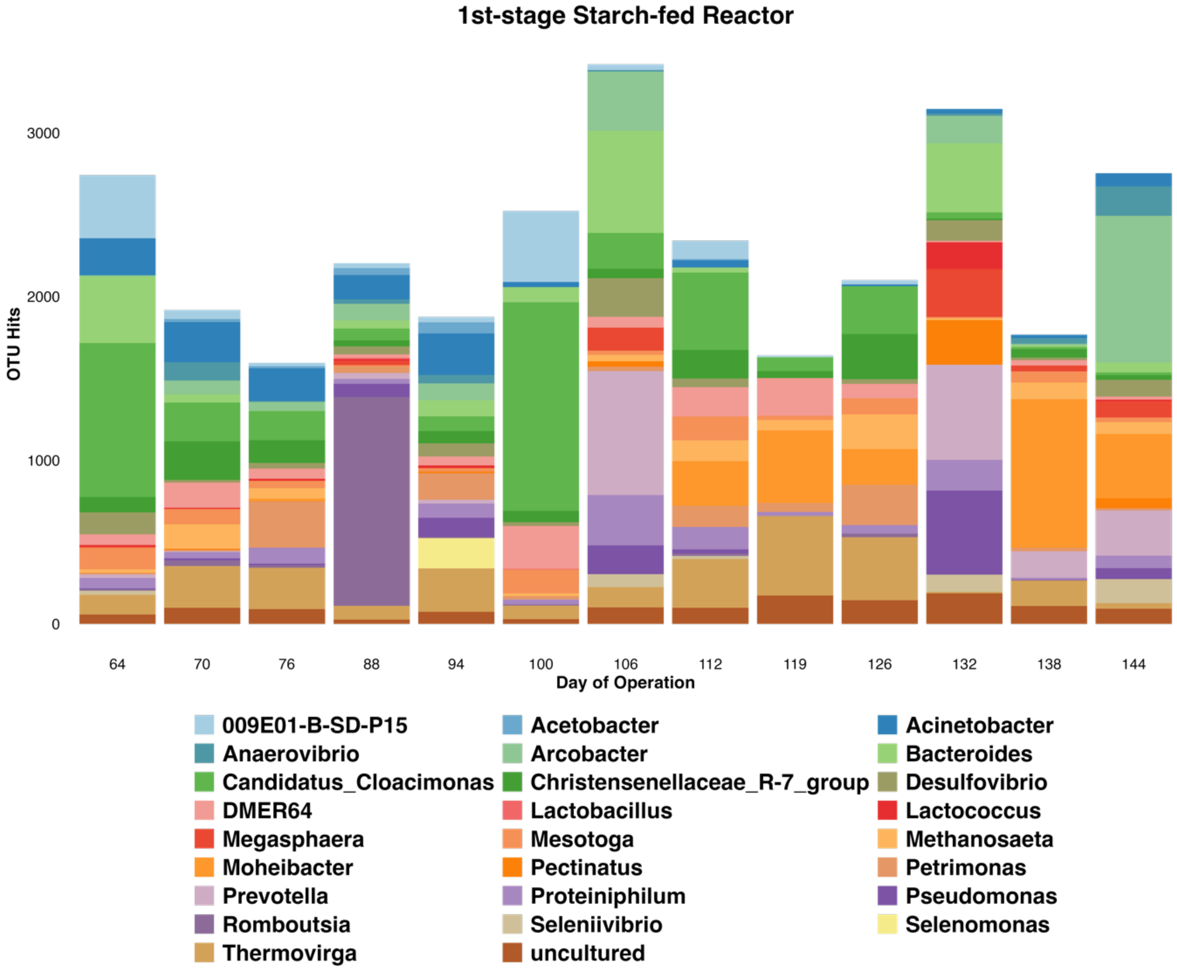
1st-stage Starch-fed Reactor
OTU Hits
0
1000
2000
3000
64
70
76
88
94
100
106
112
119
126
132
138
144
Day of Operation
009E01-B-SD-P15
Acetobacter
Acinetobacter
Anaerovibrio
Arcobacter
Bacteroides
Candidatus_Cloacimonas
Christensenellaceae_R-7_group
Desulfovibrio
DMER64
Lactobacillus
Lactococcus
Megasphaera
Mesotoga
Methanosaeta
Moheibacter
Pectinatus
Petrimonas
Prevotella
Proteiniphilum
Pseudomonas
Romboutsia
Seleniivibrio
Selenomonas
Thermovirga
uncultured

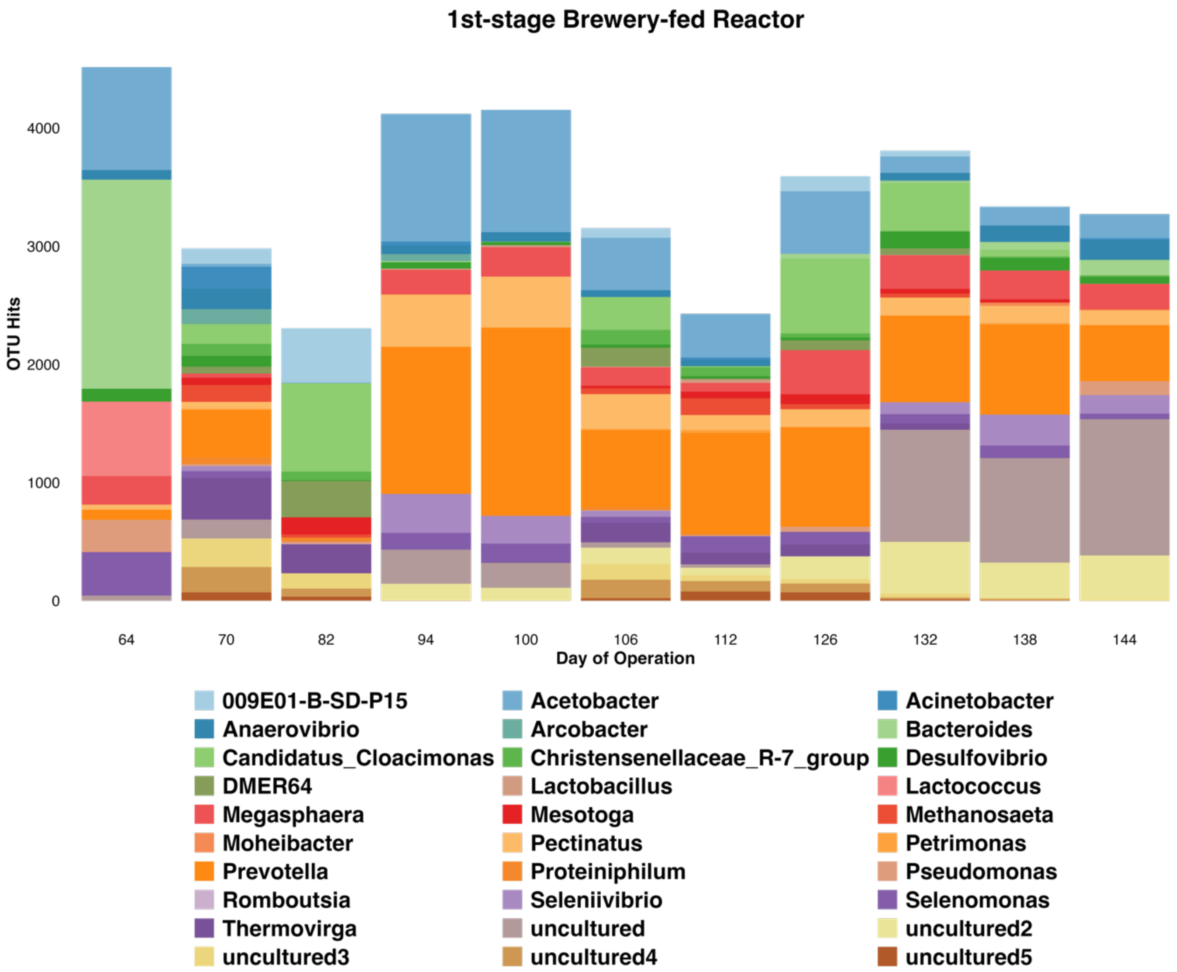
1st-stage Brewery-fed Reactor
OTU Hits
4000
3000
2000
1000
0
64
70
82
94
100
106
112
126
132
138
144
Day of Operation
009E01-B-SD-P15
Acetobacter
Acinetobacter
Anaerovibrio
Arcobacter
Bacteroides
Candidatus_Cloacimonas
Christensenellaceae_R-7_group
Desulfovibrio
DMER64
Lactobacillus
Lactococcus
Megasphaera
Mesotoga
Methanosaeta
Moheibacter
Pectinatus
Petrimonas
Prevotella
Proteiniphilum
Pseudomonas
Romboutsia
Seleniivibrio
Selenomonas
Thermovirga
uncultured
uncultured2
uncultured3
uncultured4
uncultured5

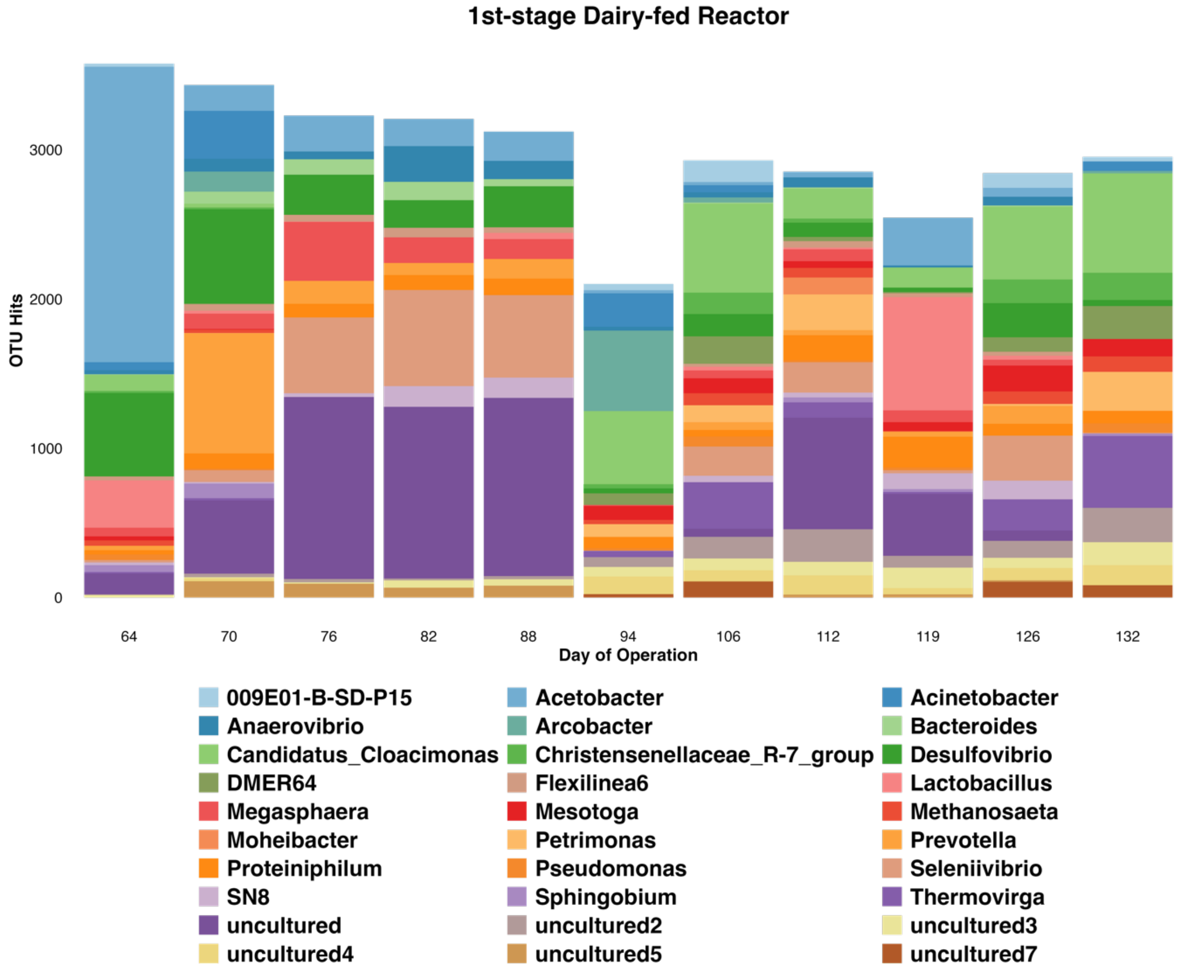
1st-stage Dairy-fed Reactor
OTU Hits
0
1000
2000
3000
64
70
76
82
88
94
106
112
119
126
132
Day of Operation
009E01-B-SD-P15
Acetobacter
Acinetobacter
Anaerovibrio
Arcobacter
Bacteroides
Candidatus_Cloacimonas
Christensenellaceae_R-7_group
Desulfovibrio
DMER64
Flexilinea6
Lactobacillus
Megasphaera
Mesotoga
Methanosaeta
Moheibacter
Petrimonas
Prevotella
Proteiniphilum
Pseudomonas
Seleniivibrio
SN8
Sphingobium
Thermovirga
uncultured
uncultured2
uncultured3
uncultured4
uncultured5
uncultured7

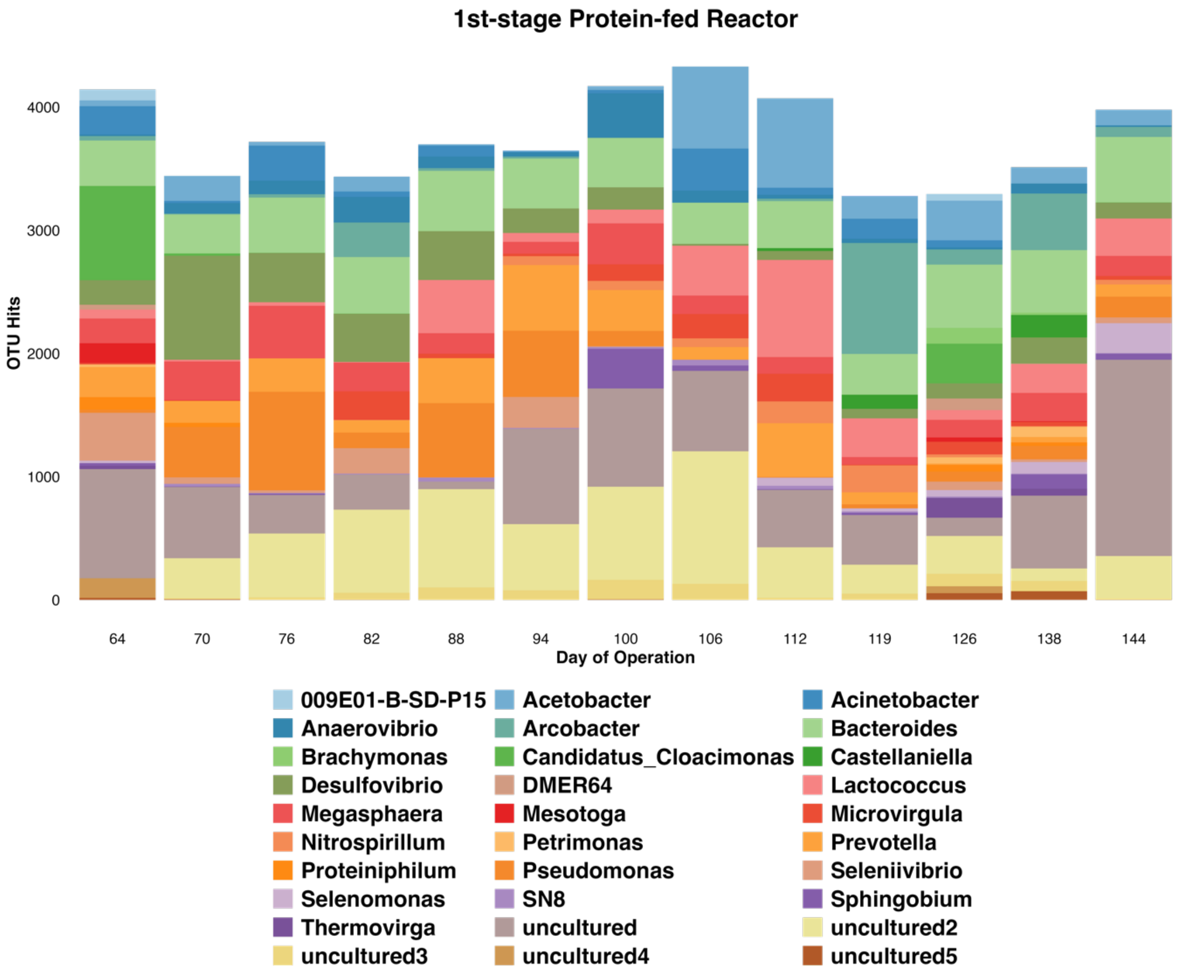
1st-stage Protein-fed Reactor
OTU Hits
0
1000
2000
3000
4000
64
70
76
82
88
94
100
106
112
119
126
138
144
Day of Operation
009E01-B-SD-P15
Acetobacter
Acinetobacter
Anaerovibrio
Arcobacter
Bacteroides
Brachymonas
Candidatus_Cloacimonas
Castellaniella
Desulfovibrio
DMER64
Lactococcus
Megasphaera
Mesotoga
Microvirgula
Nitrospirillum
Petrimonas
Prevotella
Proteiniphilum
Pseudomonas
Seleniivibrio
Selenomonas
SN8
Sphingobium
Thermovirga
uncultured
uncultured2
uncultured3
uncultured4
uncultured5

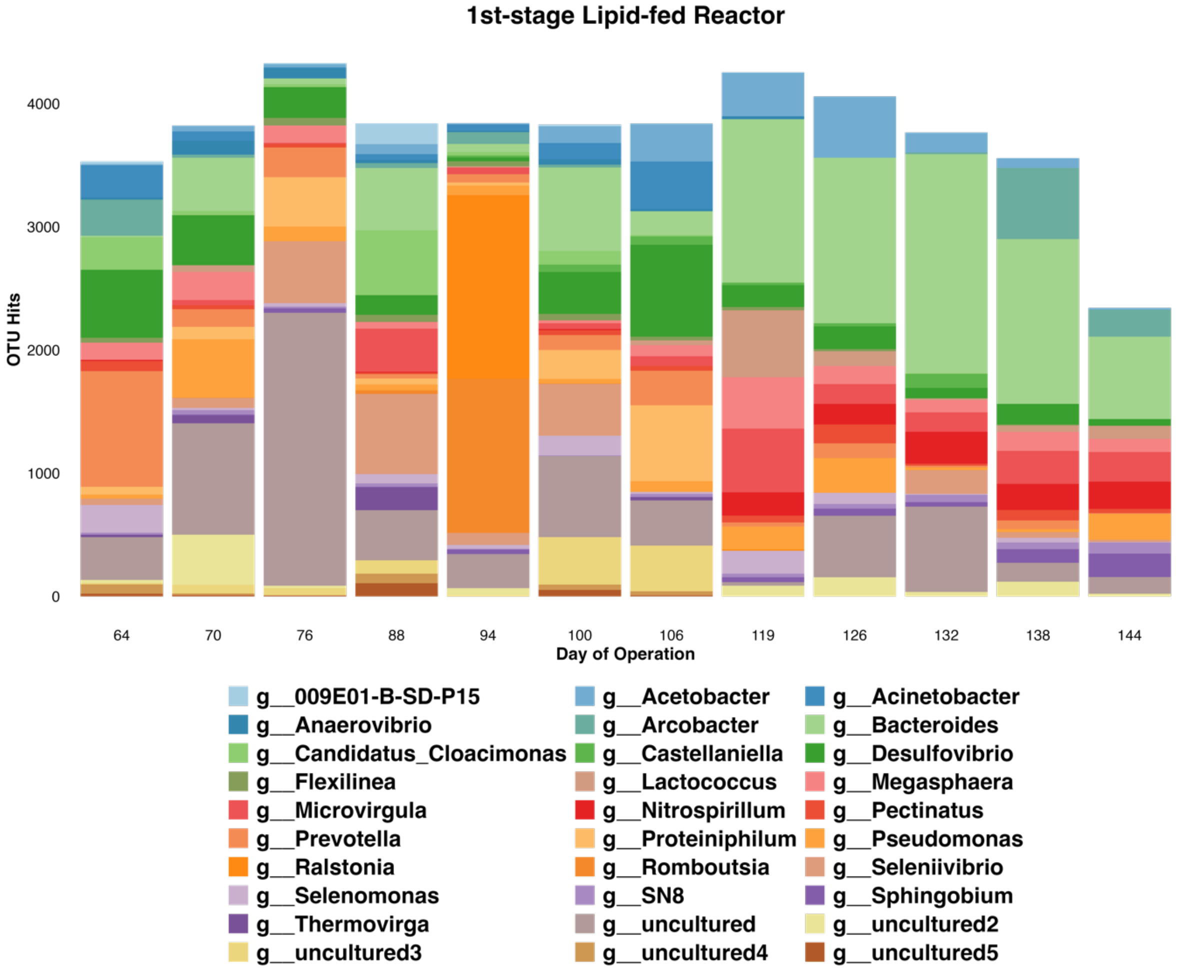

Figure S5. The top 30 OTUs identified in the 1st-stage reactors fed the five different feeds.

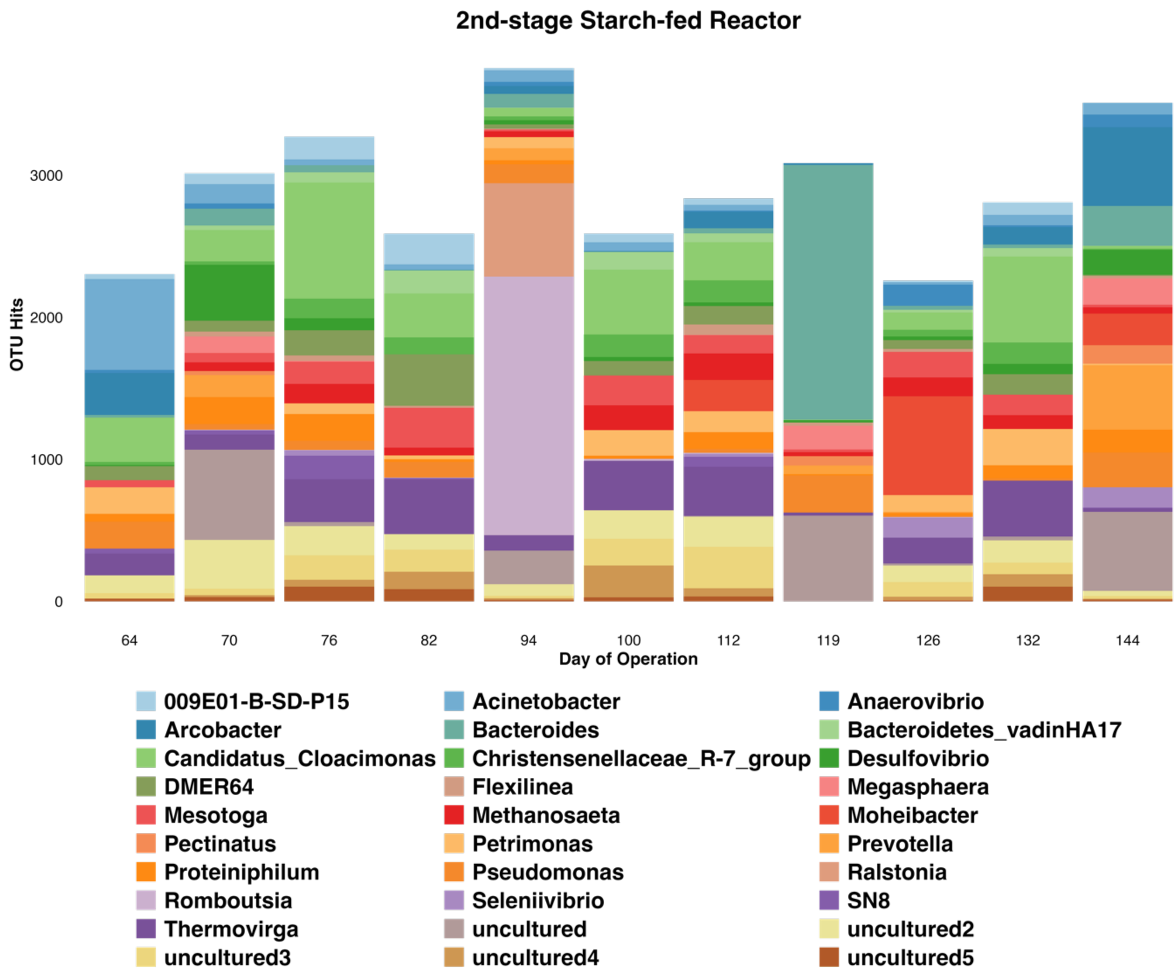
2nd-stage Starch-fed Reactor
OTU Hits
0
1000
2000
3000
64
70
76
82
94
100
112
119
126
132
144
Day of Operation
009E01-B-SD-P15
Acinetobacter
Anaerovibrio
Arcobacter
Bacteroides
Bacteroidetes_vadinHA17
Candidatus_Cloacimonas
Christensenellaceae_R-7_group
Desulfovibrio
DMER64
Flexilinea
Megasphaera
Mesotoga
Methanosaeta
Moheibacter
Pectinatus
Petrimonas
Prevotella
Proteiniphilum
Pseudomonas
Ralstonia
Romboutsia
Seleniivibrio
SN8
Thermovirga
uncultured
uncultured2
uncultured3
uncultured4
uncultured5

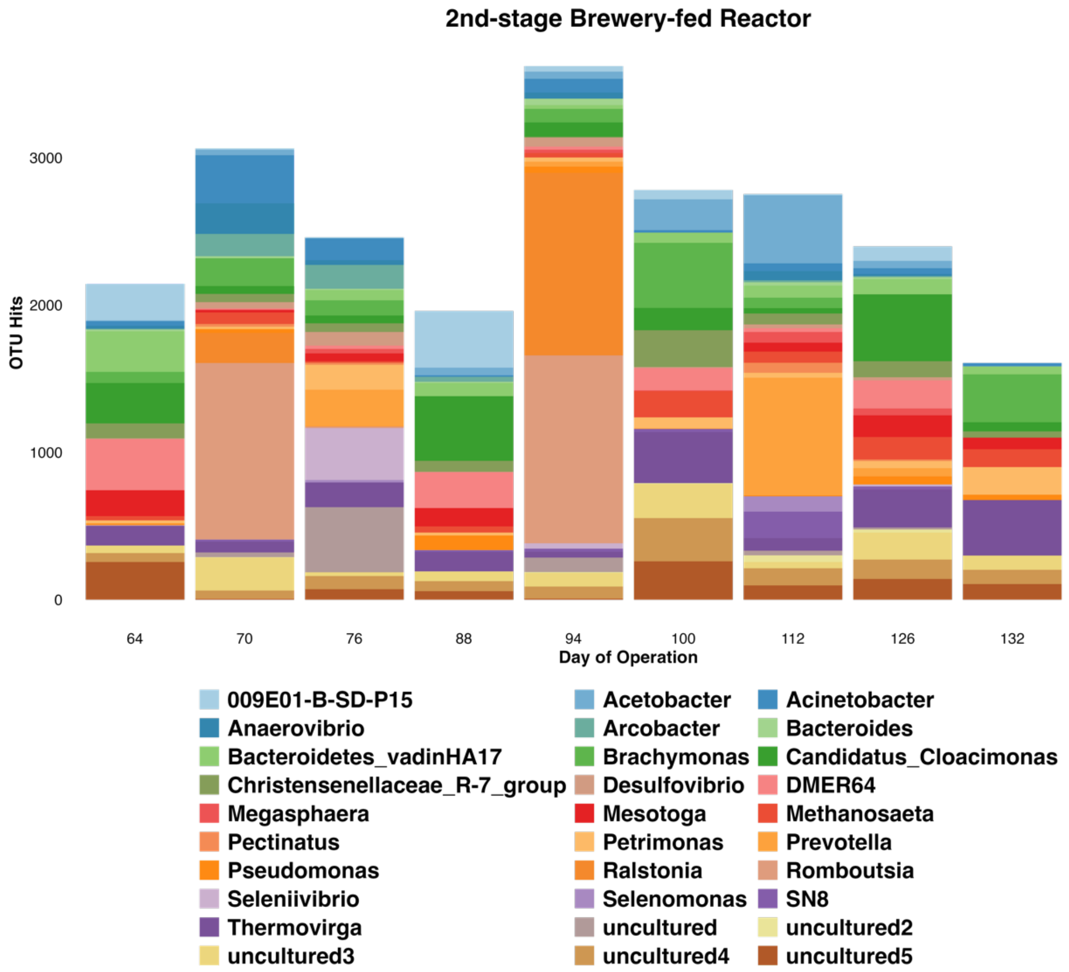
2nd-stage Brewery-fed Reactor
OTU Hits
3000
2000
1000
0
64
70
76
88
94
100
112
126
132
144
Day of Operation
009E01-B-SD-P15
Acetobacter
Acinetobacter
Anaerovibrio
Arcobacter
Bacteroides
Bacteroidetes_vadinHA17
Brachymonas
Candidatus_Cloacimonas
Christensenellaceae_R-7_group
Desulfovibrio
DMER64
Megasphaera
Mesotoga
Methanosaeta
Pectinatus
Petrimonas
Prevotella
Pseudomonas
Ralstonia
Romboutsia
Seleniivibrio
Selenomonas
SN8
Thermovirga
uncultured
uncultured2
uncultured3
uncultured4
uncultured5

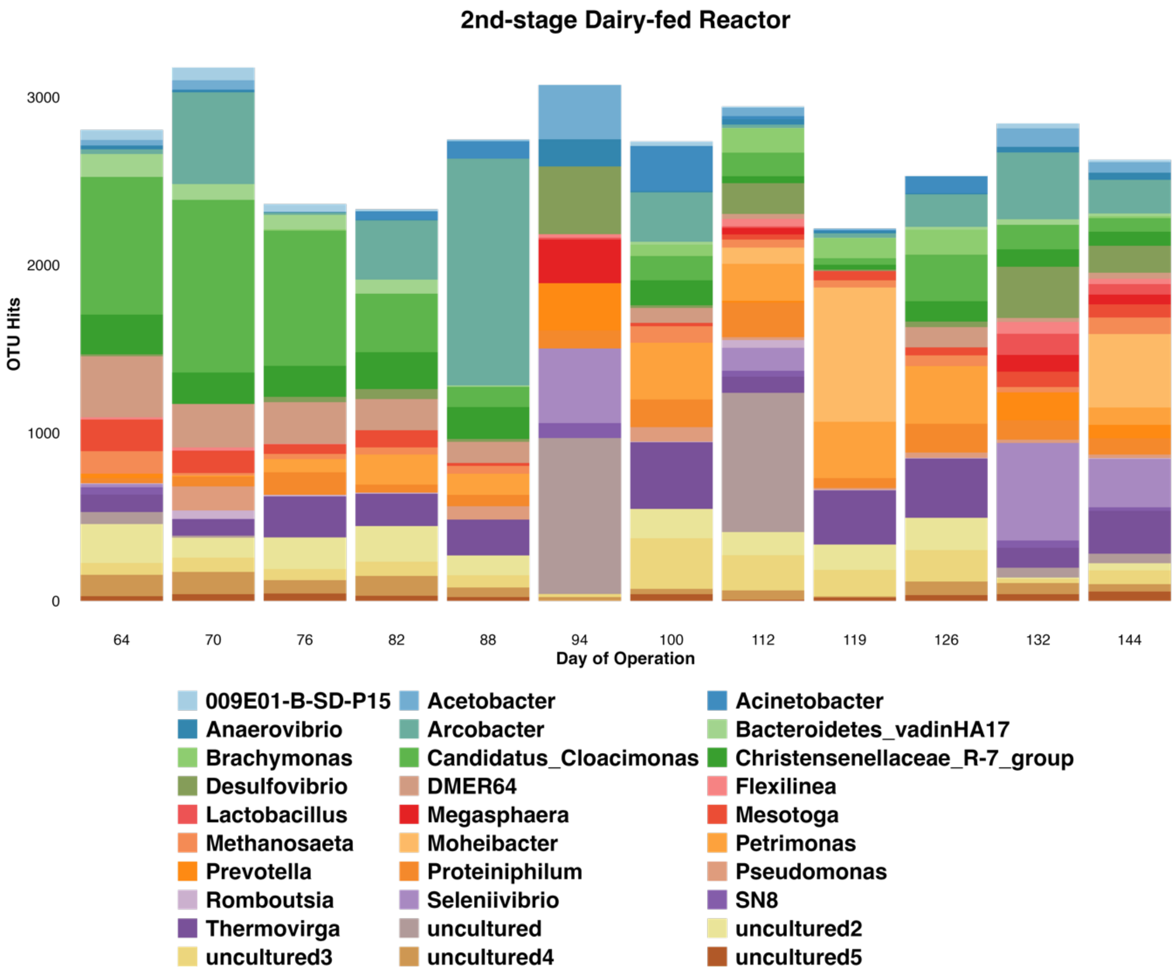

2nd-stage Dairy-fed Reactor
OTU Hits
3000
2000
1000
0
64
70
76
82
88
94
100
112
119
126
132
144
Day of Operation
009E01-B-SD-P15
Acetobacter
Acinetobacter
Anaerovibrio
Arcobacter
Bacteroidetes_vadinHA17
Brachymonas
Candidatus_Cloacimonas
Christensenellaceae_R-7_group
Desulfovibrio
DMER64
Flexilinea
Lactobacillus
Megasphaera
Mesotoga
Methanosaeta
Moheibacter
Petrimonas
Prevotella
Proteiniphilum
Pseudomonas
Romboutsia
Seleniivibrio
SN8
Thermovirga
uncultured
uncultured2
uncultured3
uncultured4
uncultured5

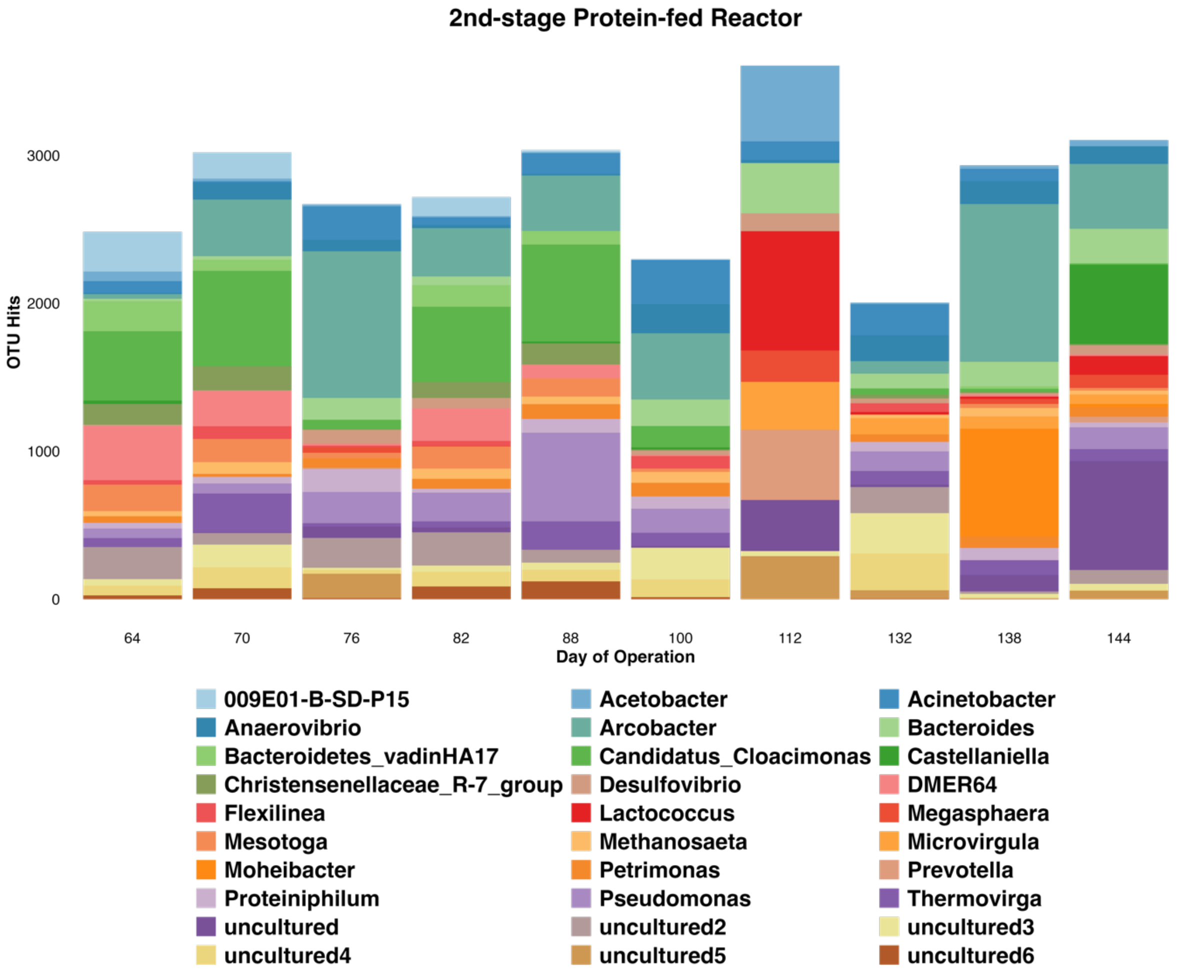
2nd-stage Protein-fed Reactor
OTU Hits
0
1000
2000
3000
64
70
76
82
88
100
112
132
138
144
Day of Operation
009E01-B-SD-P15
Acetobacter
Acinetobacter
Anaerovibrio
Arcobacter
Bacteroides
Bacteroidetes_vadinHA17
Candidatus_Cloacimonas
Castellaniella
Christensenellaceae_R-7_group
Desulfovibrio
DMER64
Flexilinea
Lactococcus
Megasphaera
Mesotoga
Methanosaeta
Microvirgula
Moheibacter
Petrimonas
Prevotella
Proteiniphilum
Pseudomonas
Thermovirga
uncultured
uncultured2
uncultured3
uncultured4
uncultured5
uncultured6

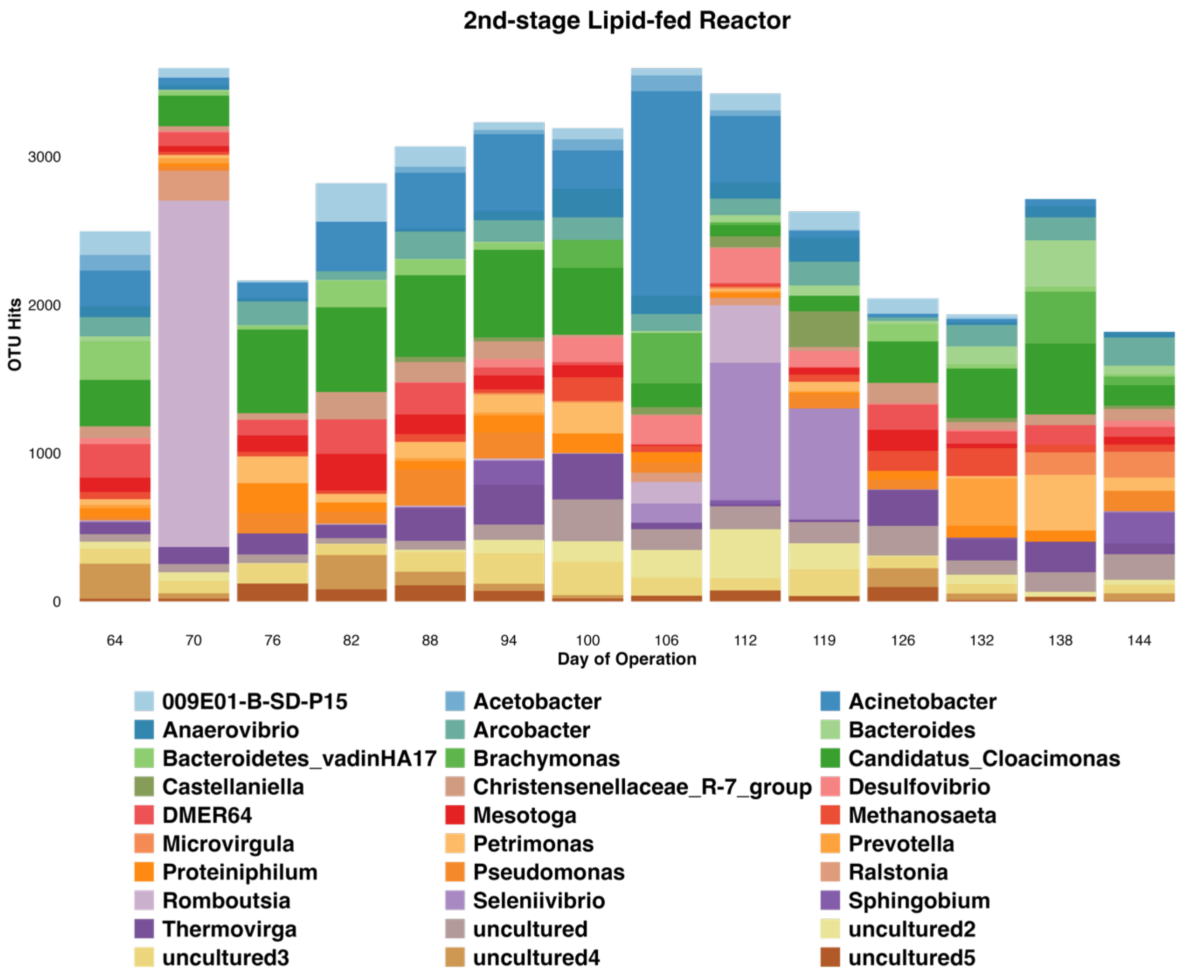

Figure S6. Top 30 OTUs identified in the 2nd-stage reactors receiving effluent from the 1st-stage reactors.

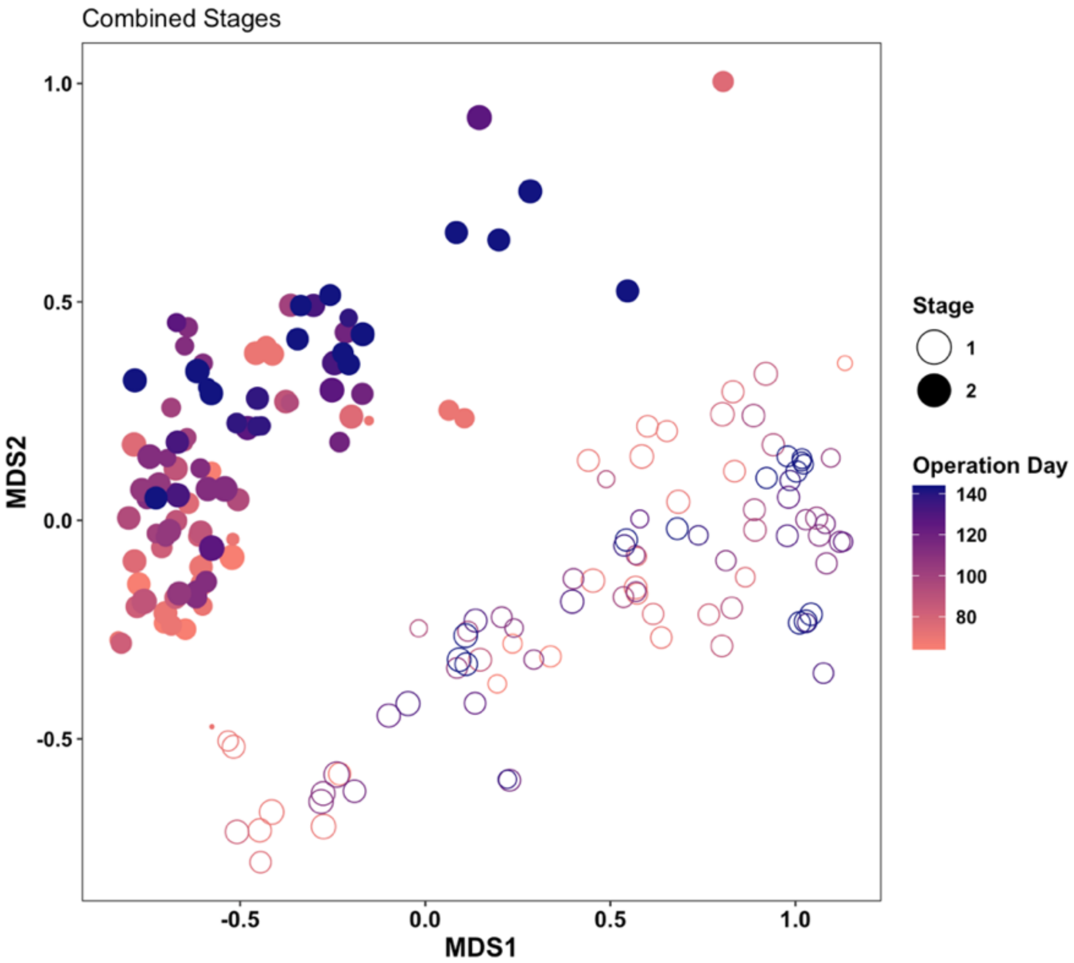

Figure S7. Non-multidimensional Scaling (MDS) using Bray-Curtis dissimilarity, showing the differences in the microbial community structure in both reactor stages over time.

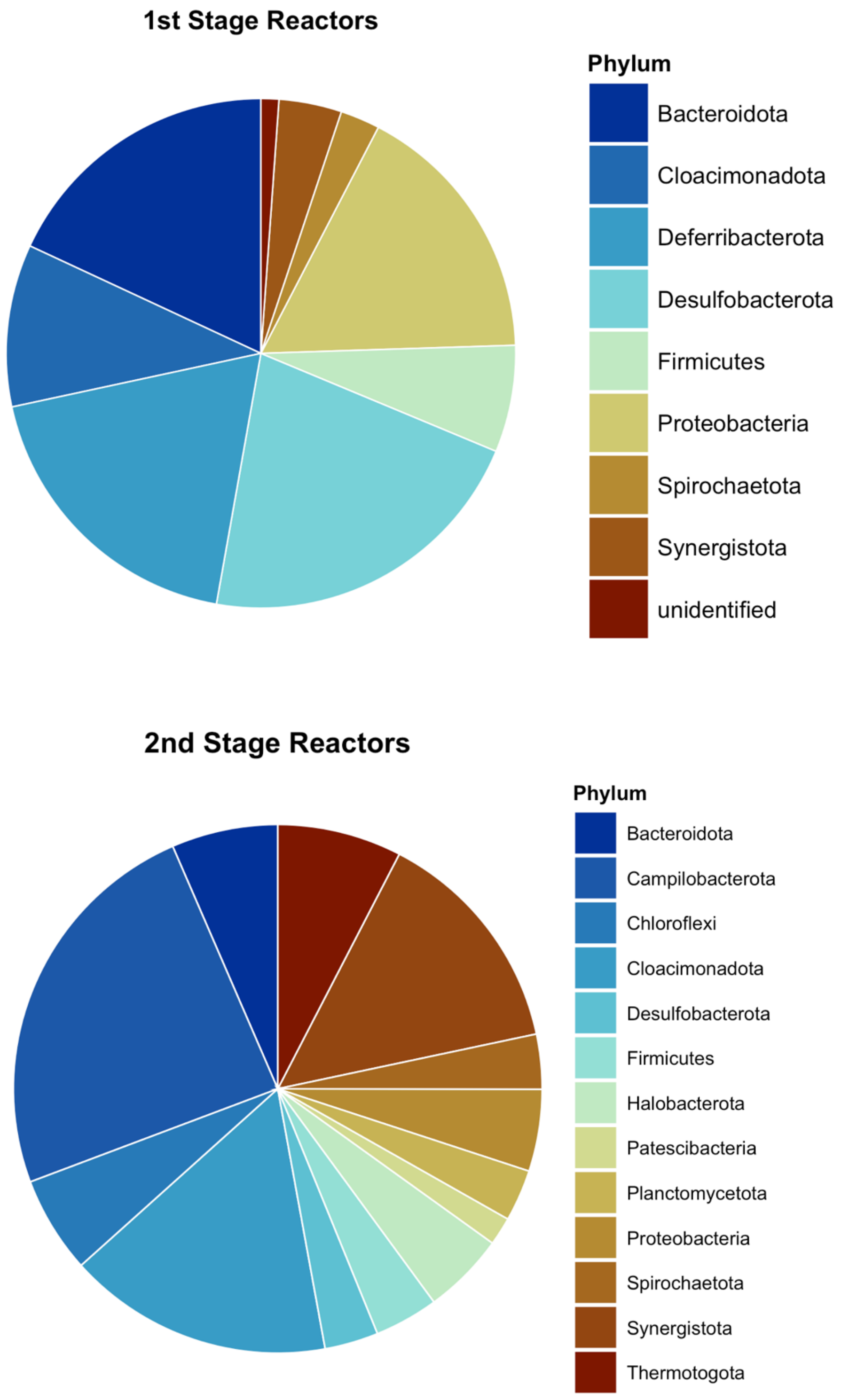

Figure S8. Core communities on the phylum level common to all feeds in the (a) 1st-stage and (b) the 2nd-stage reactor samples.

Table S2. Core genera identified in the 1st and 2nd-stage reactors

1st -stage Core Genera	2nd-stage Core Genera
Uncultured	Uncultured
Prevotella	*Bacteroides*
Bacteroides	*Acetobacter*
Acetobacter	*Candidatus* Cloacimonas
Candidatus Cloacimonas	*Pseudomonas*
Pseudomonas	*Arcobacter*
Desulfovibrio	*Desulfovibrio*
Megasphaera	*Thermovirga*
Uncultured	Uncultured
Uncultured	*Acinetobacter*
Seleniivibrio	Uncultured
Lactococcus	DMER64
Proteiniphilum	*Proteiniphilum*
Anaerovibrio	*Anaerovibrio*
Pectinatus	*Christensenellaceae* R-7 group
Uncultured	*Petrimonas*
SN8	*Mesotoga*
Nitrospirillum	*Methanosaeta*
Macellibacteroides	009E01-B-SD-P15
Acidaminococcus	Uncultured
UCG-009	Uncultured
Unidentified_1	*Flexilinea*
Unidentified_2	W5
Unidentified_3	*Macellibacteroides*
	Fluviicola
	Fermentimonas
	Sphaerochaeta
	Dechlorobacter
	Leptolinea

	Lentimicrobium
	Sedimentibacter
	Methanospirillum
	Uncultured
	Syner-01
	Saccharimonadales
	Thauera
	Pirellula
	[Eubacterium] coprostanoligenes group
	Uncultured
	RBG-13-54-9
	Dysgonomonadaceae
	Devosia
